# Comprehensive analysis of prognosis of cuproptosis-related oxidative stress genes in multiple myeloma

**DOI:** 10.3389/fgene.2023.1100170

**Published:** 2023-03-31

**Authors:** Tingting Li, Lan Yao, Yin Hua, Qiuling Wu

**Affiliations:** Institute of Hematology, Union Hospital, Tongji Medical College, Huazhong University of Science and Technology, Wuhan, China

**Keywords:** multiple myeloma, prognosis, cuproptosis, oxidative stress, overall survival

## Abstract

**Introduction:** Multiple myeloma (MM) is a highly heterogeneous hematologic malignancy. The patients’ survival outcomes vary widely. Establishing a more accurate prognostic model is necessary to improve prognostic precision and guide clinical therapy.

**Methods:** We developed an eight-gene model to assess the prognostic outcome of MM patients. Univariate Cox analysis, Least absolute shrinkage and selection operator (LASSO) regression, and multivariate Cox regression analyses were used to identify the significant genes and construct the model. Other independent databases were used to validate the model.

**Results:** The results showed that the overall survival of patients in the high-risk group was signifificantly shorter compared with that of those in the low-risk group. The eight-gene model demonstrated high accuracy and reliability in predicting the prognosis of MM patients.

**Discussion:** Our study provides a novel prognostic model for MM patients based on cuproptosis and oxidative stress. The eight-gene model can provide valid predictions for prognosis and guide personalized clinical treatment. Further studies are needed to validate the clinical utility of the model and explore potential therapeutic targets.

## 1 Introduction

Multiple myeloma, the second most common hematologic malignancy, accounts for 1.8% of all malignancies. It is a heterogeneous malignant plasma cell disorder characterized by aberrant proliferation of mature B cells (Mitsiades et al., 2007; Rajkumar et al., 2014). The first-line treatment for multiple myeloma (MM) mainly consists of the combination of bortezomib, and dexamethasone with either cyclophosphamide or doxorubicin. With the introduction of more effective proteasome inhibitors, such as carfilzomib and more potent immunomodulatory drugs such as pomalidomide, as well as the development of a new class of monoclonal antibody therapies, the clinical outcome of relapsed or refractory MM patients has improved significantly (Joshua et al., 2019). However, MM remains incurable with a high recurrence rate. The prognosis of MM patients is still poor (Palumbo and Anderson, 2011; Sonneveld et al., 2016). Therefore, performing risk stratification for patients and finding a new prognostic biomarker is crucial to improve prognostic accuracy and direct therapeutic treatment.

Copper ion plays a crucial role in numerous biological processes, including mitochondrial respiration, redox signaling, kinase signaling, cell wall remodeling, oxidative stress responses and other processes (Rae et al., 1999; Kim et al., 2008; Yruela, 2009). Dysregulation of copper plays a key role in cancer and mitochondria in many diseases. Cuproptosis is a recently identified form of programmed cell death (PCD) distinguished from known death mechanisms like apoptosis necroptosis, pyroptosis, and ferroptosis. Cuproptosis occurs through the binding of the intracelluar copper to lipoylated components of the tricarboxylic acid (TCA) cycle in mitochondria (Tsvetkov et al., 2022). This leads to aggregation of lipoylated protein and subsequent loss of Fe-S cluster-containing proteins, which results in acute proteotoxic stress and ultimately cell death. Although cuproptosis has received increased attention, the mechanism and role of cuprotosis in multiple myeloma remain unclear.

Oxidative stress occurs due to the excessive production of reactive oxygen species (ROS) or the failure of antioxidants to eliminate ROS inadequately. It is an essential factor in driving tumorigenesis and cancer progression (Schieber and Chandel, 2014; Lipchick et al., 2016). The production and degradation of ROS levels are strictly controlled in normal cells. Mitochondria are thought to be primary sources of ROS (Holmström and Finkel, 2014). The dysregulation of ROS induces mitochondrial DNA damage (Cadenas and Davies, 2000). Additionally, accumulating DNA damage eventually results various genomic alterations and initiates tumorigenesis. Only a few studies describe genetic events in multiple myeloma cells that primarily affect intracellular redox status during its progression (Lipchick et al., 2016). ACA11, an orphan box H/ACA class small nucleolar RNA, was shown to inhibit oxidative stress in multiple myeloma cell, which was upregulated in MM patient with t (Palumbo and Anderson, 2011; Lipchick et al., 2016) chromosomal translocation (Chu et al., 2012). The relationships between oxidative stress with survival in MM patients needs further investigation.

The hypothesis of this study is that combining cuproptosis and oxidative stress can provide a more accurate prognostic value for patients with multiple myeloma (MM). To test this hypothesis, we developed a novel 8-cuproptisis-associated-oxidative stress signature to predict the survival outcomes of MM patients by analyzing data obtained from the Gene Expression Omnibus database. This signature was helpful for risk stratification and prognosis. Furthermore we established a prognostic nomogram that could accurately predict overall survival of MM patients. Our findings may shed light on the underlying mechanisms of MM progression and suggest that the signature may serve as a promising prognostic marker for multiple myeloma patients.

## 2 Materials and methods

### 2.1 Data acquisition

Gene expression profiles were downloaded from the Gene Expression Omnibus (GEO) database (http://www.ncbi.nlm.nih.gov/gds/). GSE24080, GSE4581 and GSE2658 were obtained using the GPL570 platform (Hanamura et al., 2006; Shi et al., 2010). GSE6477 was obtained using the GPL96 platform (Chng et al., 2007). GS136337 was obtained using the GPL27143 platform (Danziger et al., 2020). Multiple Myeloma Research Foundation (MMRF) CoMMpass study offers the expression profiles of MM patients and clinical information (including survival statistics). The normalized was conducted by the “limma” R package.

Thirteen cuproptosis-related genes were obtained from previous studies (Supplementary Table S1) (Tsvetkov et al., 2022) (Oliveri, 2022; Tang et al., 2022). 1,399 oxidative stress-related genes were extracted from GeneCards (https://www.genecards.org) with a relevance score ≥ 7 (Supplementary Table S2) (Wu et al., 2021).

**FIGURE 1:**
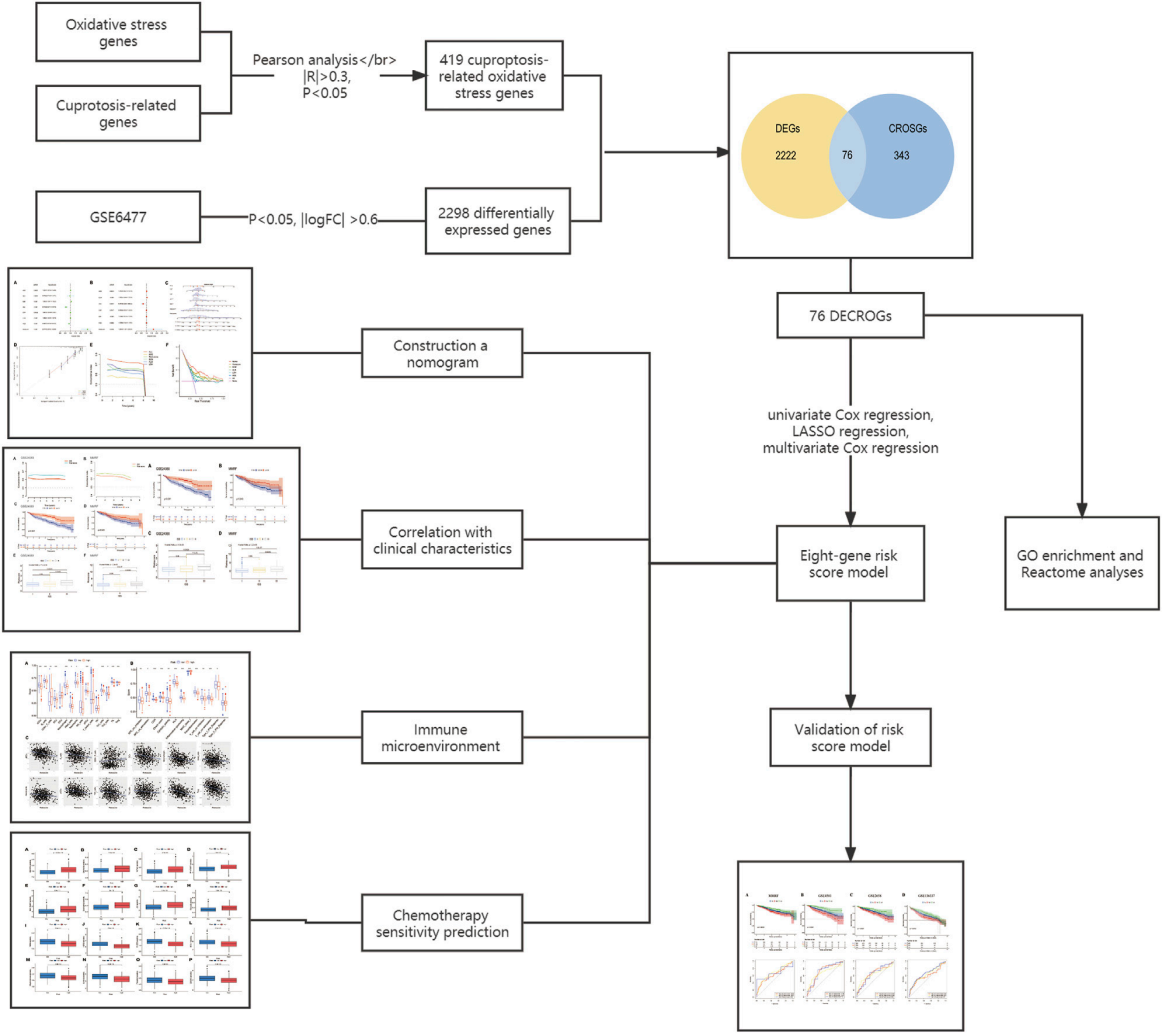
Flow chart of this study.

The flowchart for this study was shown in Figure 1.

### 2.2 Cuproptosis -related oxidative stress genes

Using Pearson correlation analysis, cuproptosis -related oxidative stress genes were identified and a co-expression network was created based on the cutoff point (Pearson |R| > 0.3 and *p* < 0.05). A total of 419 cuproptosis-related oxidative stress genes were identified.

### 2.3 Differential gene expression analysis

With *p* < 0.05 and |log2 Fold Change|≥ 0.6 as cut-off points, differentially expressed genes (DEGs) between MM patients and normal donors were identified using the “limma” package in R language. To illustrate DEGs, heat maps and volcano were created using the R package “ggplot2” and “pheatmap”. We then took the intersection of DEGs and cuproptosis-related oxidative stress genes to obtain differentially expressed cuproptosis -related oxidative stress genes (DECROGs).

### 2.4 Functional enrichment analysis of DECROGs

For the DECROGs, the gene ontology (GO) annotation and Reactome pathway analysis were applied to investigated their functions and pathway enrichment utilizing the R package clusterProfiler (Yu et al., 2012). The cutoff criterion was the false-discovery rate (FDR). Terms with FDR <0.05 represented significant enrichment. GO enrichment consists of three components: biological process (BP), molecular function (MF) and cellular component (CC).

### 2.5 Construction and validation of a prognostic risk model

We identified prognosis-related genes by the univariate Cox regression analysis (*p* < 0.05). These genes were entered into the Least Absolute Shrinkage and Selection Operator (LASSO) regression analysis to screen key genes (*p* < 0.05) through the R package glmnet (Friedman et al., 2010). Then, multivariate Cox regression was used to analyzed these genes and establish the risk score model. Risk scores of all samples were calculated according to the following formula: risk score = ∑Coef* Exp. The “Coef” refers to the coefficient of each mRNA from the multivariate Cox analysis and “Exp” indicates the expression level of each mRNA. We validated the risk model using the GSE2658, GSE136337, GSE4581 and MMRF datasets. The median risk score was used as the cut-off value to divide the high-risk and low-risk groups, The difference in overall survival between the high- and low-risk groups was evaluated using the Kaplan-Meier survival analysis and log-rank test. The R package “survivalROC” was utilised to produce the time-dependent Receiver Operating Characteristic (ROC) curves.

### 2.6 Construction and evaluation of a predictive nomogram

We constructed a nomogram prognostic prediction model based on risk scores and clinical pathological feature using the “rms” R package. Concordance index (C-index), calibration curve, and Decision Curve Analysis (DCA) were used to assess the prognostic performance of the established nomogram.

### 2.7 Immune microenviroment

Immune infiltration analysis was analyzed by single-sample gene set enrichment analysis (ssGSEA) method. This involved the enrichment scores of 16 immune cell and 13 immune function categories. The “GSVA” package was utilized for the analysis (Hänzelmann et al., 2013). The correlation between the immune cell scores and the signature was investigated by Pearson correlation analysis, and the “corrplot” package was used for the analysis.

### 2.8 Drug sensitivity analysis

The data on drug sensitivity was obtained from the Genomics of Drug Sensitivity in Cancer (GDSC) database (Yang et al., 2013). “OncoPredict” package was used to calculate the 50% inhibiting concentration (IC50) values in the high- and low-risk groups (Maeser et al., 2021).

### 2.9 Statistical analyses

R software (version 4.2.0) and corresponding packages were carried out for statistical analyses. The Kaplan-Meier method and log-rank test were used to assess prognosis. Pearson method was applied for correlation analysis. The Wilcoxon test was used to compare data between two groups, while the Kruskal-Wallis H test was used for data comparison among three groups. All tests with *p*-value <0.05 indicated statistical significance.

## 3 Results

### 3.1 DECROGs identification and functional enrichment analysis

**FIGURE 2:**
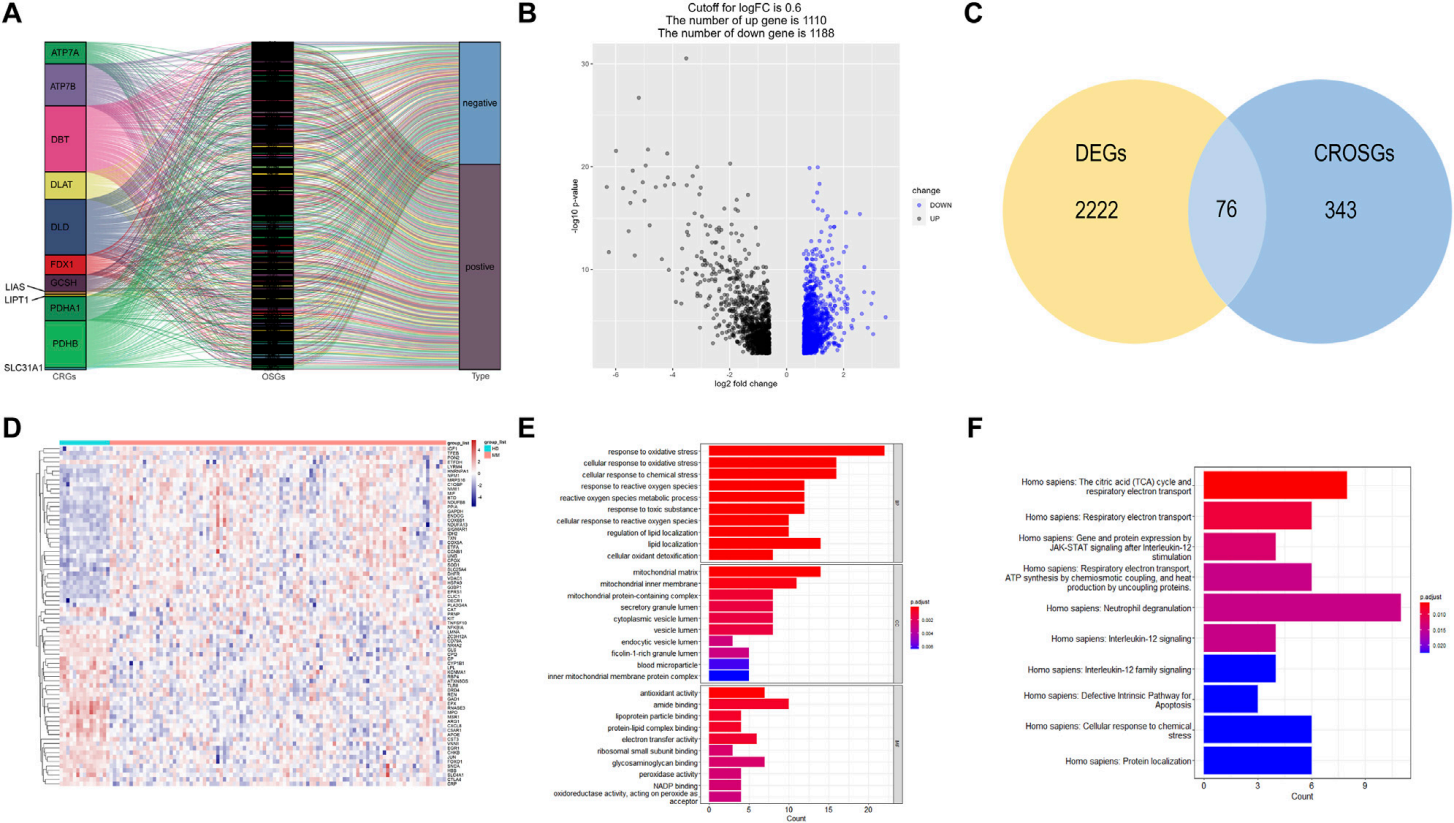
Identification of candidate DECROGs. **(A)** Sankey diagram showed the correlations between CRGs and OSGs **(B)**The volcano plot between healthy donors and MM patients. **(C)** Venn diagram identifying DEGs correlated with cuproptosis-related oxidative stress genes. **(C)** Heatmap of the expressions of 76 DECROGs. **(E)** Top 10 classes of GO enrichment terms in biological process (BP), cellular component (CC), and molecular function (MF). **(F)** Top 10 classes of Reactome pathway enrichment terms.

The correlation between cuproptosis-related mRNAs and oxidative stress genes was analyzed by the Pearson correlation coefficient method (Pearson |R| > 0.3 and *p* < 0.05). A total of 419 cuproptosis-related oxidative stress genes were identified. The sankey plot demonstrated the correlation between cuproptosis-related genes (CRGs) and oxidative stress genes (OSGs) (Figure 2A). Then, we set a *p* < 0.05, and [log2FoldChange (log2FC)] > 0.6 as the cutoff point. Based on this criteria, we identified 2,298 DEGs in the GSE6477 dataset comparing multiple myeloma with healthy donors. The volcano plot of DEGs was illustrated in Figure 2B. Finally, 2,298 DEGs were intersected with 419 cuproptosis-related oxidative stress genes and thus we obtained 76 differentially expressed cuproptosis-related oxidative stress genes (DECROGs). The Venn diagram was shown in Figure 2C and the heatmap of the 76 DECROGs are shown in Figure 2D.

GO enrichment and Reactome analyses were conducted for exploring the potential function of 76 DECROGs. The result was demonstrated in Figures 2E, F. The results of GO enrichment analysis were categorized into the following three parts. For biological process (BP), DECROSGs were enriched in response to oxidative stress, and lipid localization. For cellular component (CC), the genes were associated with lumen and membrane. For molecular function (MF), the genes were associated with antioxidant activity and lipid-protein binding. Reactome pathway analysis demonstrated that the DECROGs were mainly associated with cell respiratiory electron transport, interleukin-12 signaling and neutrophil degranulation, defective intrinsic pathway for apoptosis.

### 3.2 Exploration of the prognostic DECROGs in MM

**FIGURE 3:**
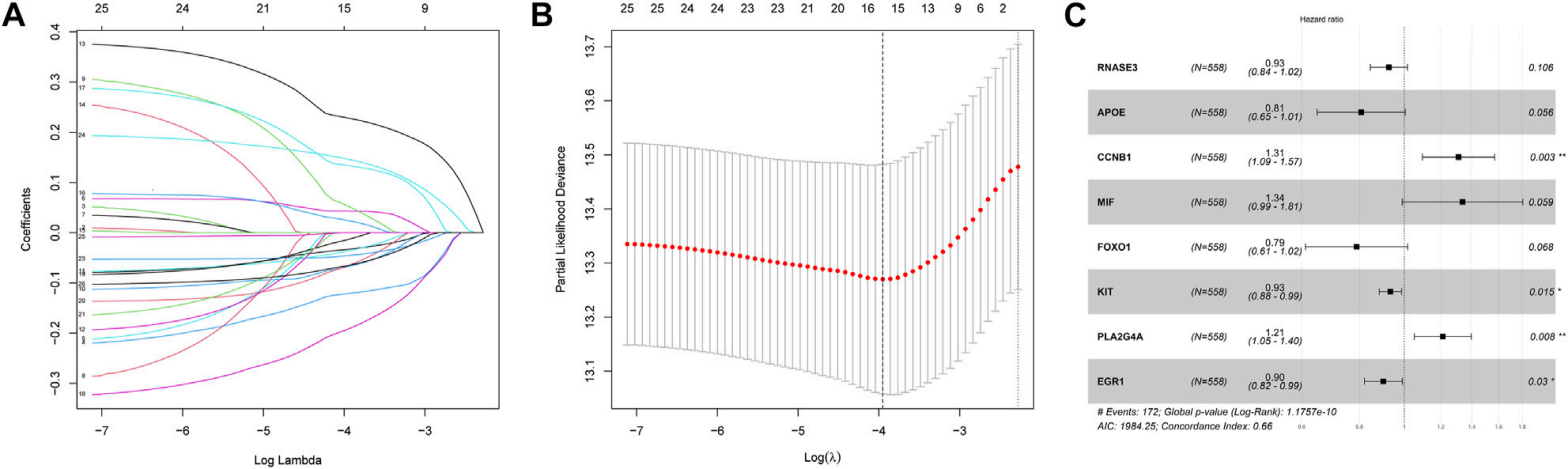
LASSO regression analysis of 26 genes. **(A)** LASSO regression analysis of 26 genes. **(B)** Cross-validation of results of LASSO regression analysis. **(C)** Multivariable Cox regression analysis of genes determined by lasso regression analysis.

Then, we investigated the prognostic significance of the 76 genes using univariate Cox regression. Consequently, a total of 26 DECROGs associated with prognosis were chosen (*p* < 0.05) (Table 1). Subsequently, the 26 DECROGs described above were integrated into the LASSO regression model (Figures 3A, B). Then stepwise multivariate Cox proportional hazard regression analysis was used and eight genes were identified to construct a prognostic model for MM patients (Figure 2C). We obtained eight genes, including *RNASE3*, *APOE*, *CCNB1*, *MIF*, *FOXO1*, *KIT*, *PLA2G4A*, *ECG1* to build the risk model for MM patients. Among them *CCNB1*, *MIF*, *PLA2G4A* may be regarded as oncogenes, whereas *RNASE3*, *APOE*, *FOXO1*, *KIT*, and *EGR1* may be tumor suppressor genes. The formula for calculating out each patient’s risk score is as follows:

$$\begin{array}{ll} Riskscore & =\mathrm{EXP}\left(RNASE3\right)*\left(-0.077\right)+\mathrm{EXP}\left(APOE\right)*\left(-0.214\right)+\mathrm{EXP}\left(CCNB1\right)*\left(0.270\right)+\mathrm{EXP}\left(MIF\right)*\left(0.290\right)\\ & +\mathrm{EXP}\left(FOXO1\right)*\left(-0.238\right)+\mathrm{EXP}\left(KIT\right)*\left(-0.069\right)+\mathrm{EXP}\left(PLA2G4A\right)*\left(0.192\right)+\mathrm{EXP}\left(EGR1\right)*\left(-0.104\right) \end{array}$$

**TABLE 1:** 26 potential genes based on univariate Cox regression analysis.

Gene	HR	HR.95L	HR.95H	*p*-value
*RNASE3*	0.887	0.810	0.972	0.011
*C5AR1*	0.917	0.841	1.000	0.049
*TXN*	1.325	1.061	1.653	0.013
*APOE*	0.680	0.549	0.840	0.000
*DHFR*	1.297	1.018	1.653	0.035
*SOD1*	1.560	1.169	2.083	0.003
*KCNMA1*	0.896	0.803	0.999	0.047
*VDAC1*	1.350	1.028	1.773	0.031
*PPIA*	1.575	1.104	2.247	0.012
*CYP1B1*	0.742	0.601	0.917	0.006
*HBB*	0.908	0.830	0.992	0.033
*NME1*	1.327	1.052	1.674	0.017
*CCNB1*	1.525	1.278	1.818	0.000
*ETFA*	1.361	1.040	1.780	0.025
*CD79A*	0.888	0.796	0.991	0.034
*DECR1*	1.544	1.138	2.096	0.005
*MIF*	1.644	1.210	2.235	0.001
*FOXO1*	0.649	0.511	0.823	0.000
*DRD4*	0.770	0.613	0.968	0.025
*IGF1*	0.762	0.621	0.934	0.009
*C1QBP*	1.272	1.030	1.571	0.025
*CPQ*	0.828	0.727	0.942	0.004
*KIT*	0.927	0.878	0.978	0.006
*PLA2G4A*	1.333	1.152	1.543	0.000
*CRP.1*	0.891	0.796	0.998	0.046
*EGR1*	0.893	0.817	0.977	0.013

HR: hazard ratios.

### 3.3 Establishment and validation of risk model for predicting overall survival

**FIGURE 4:**
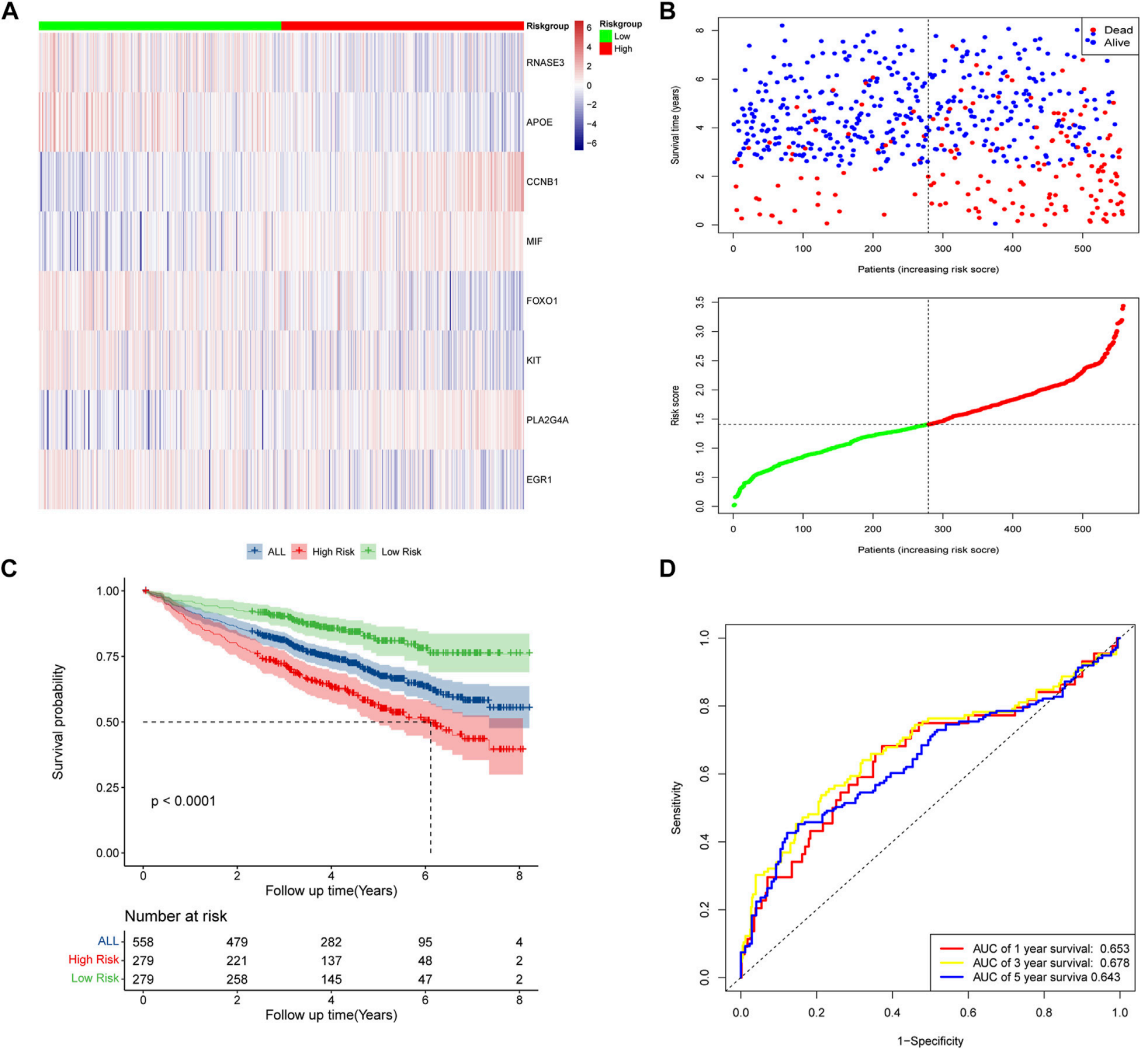
Risk score model based on eight-gene signature in GSE24080. **(A)** Gene expression heat map for eight prognostic genes in the high-risk and low-risk groups. **(B)** MM patients were divided into high-risk and low-risk groups based on the median risk score. Scatter plot of survival time and status in the high-risk and low-risk groups. **(C)** Kaplan–Meier curves of overall survival. **(D)** Assessment of the predictive ability of the model by time-dependent ROC analysis.

According to the median risk scores of 558 samples, high-risk subgroups (*n* = 279) and low-risk subgroups (*n* = 279) were stratified. The heat map showed the expression levels of the eight DECROGs in the high- and low-risk groups (Figure 4A). Figure 4B shows the distribution of risk scores and survival status between the high-risk and low-risk groups. The mortality rate of patients increased with the increasing risk score.

Kaplan-Meier analysis showed that overall survival was worse in high-risk patients than in low-risk patients (*p* < 0.0001, Figure 4C). We evaluated the efficacy of the prognostic model by time-dependent ROC curves, and the area under the curves (AUC) was 0.653 for 1-year survival, 0.678 for 3-year survival, and 0.643 for 5-year survival (Figure 4D), indicating a moderate performance of this model.

**FIGURE 5:**
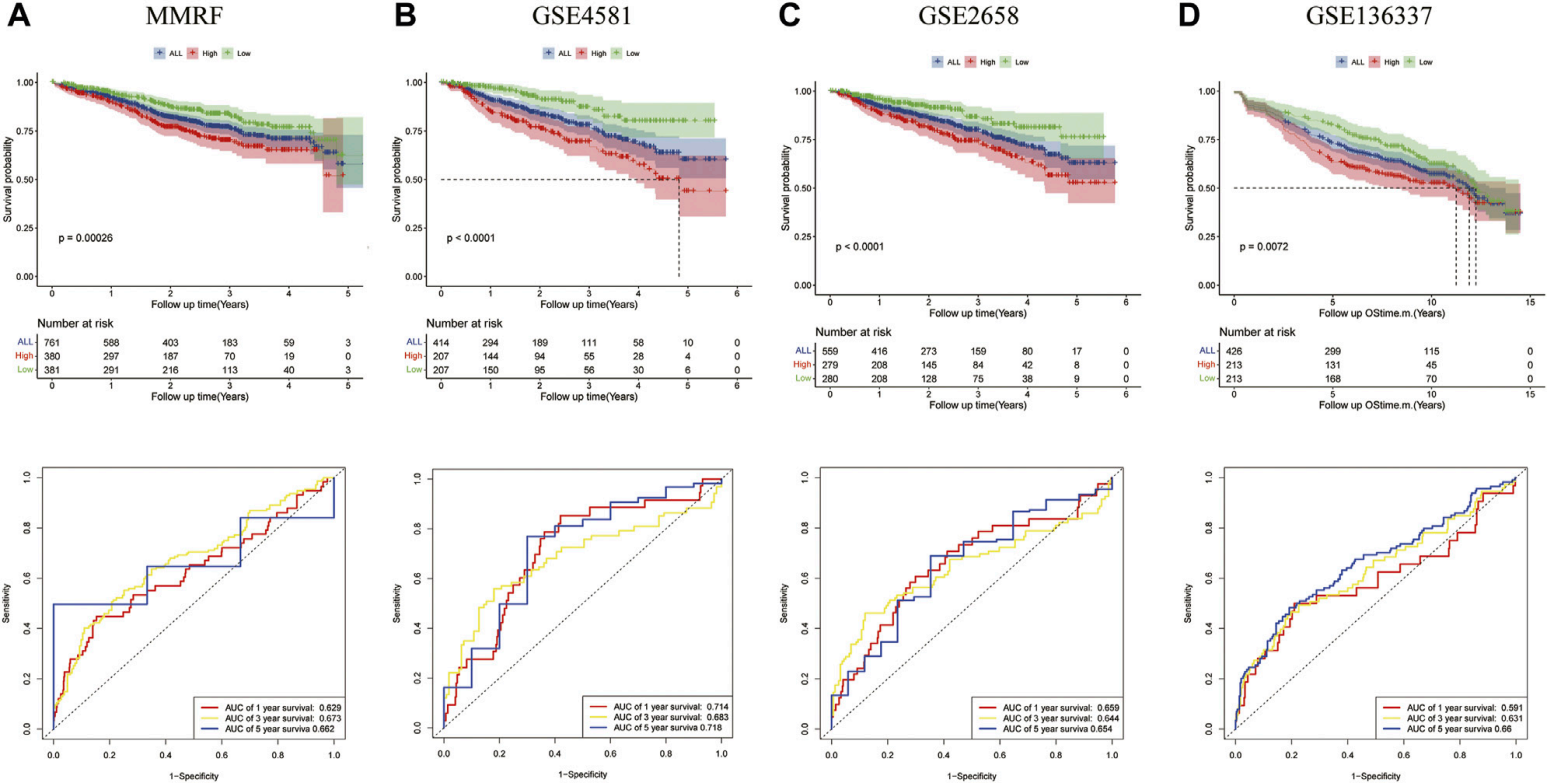
Validation of the eight-gene risk score model in the testing datasets. **(A)** The Kaplan–Meier OS curves for patients in the high- and low-risk groups in the MMRF cohort (*p* = 0.00026, top). ROC curves showed the prognostic performance of the prognostic model in the MMRF cohort. (bottom) **(B)** OS in GSE4581 (*p* < 0.0001, top), ROC curve in GSE4581 (bottom). **(C)** OS in GSE2658 (*p* < 0.0001, top), ROC curve in GSE4581 (bottom). **(D)** OS in GSE136337 (*p* = 0.0072, top), ROC curve in GSE136337 (bottom).

The prognostic significances of this 8-gene signature were further vilified in 3 external independent GEO datasets and MMRF datasets with over 2,000 MM patients (Figure 5). The results showed that the overall survival of patients in the high-risk groups was significantly worse than that of patients in the low-risk group. The AUC of the risk model was >0.6, proving the performance of this model. In summary, we established an eight-gene risk model that exhibited acceptable performance in these datasets.

### 3.4 Construction and validation of the nomogram prognostic model

**FIGURE 6:**
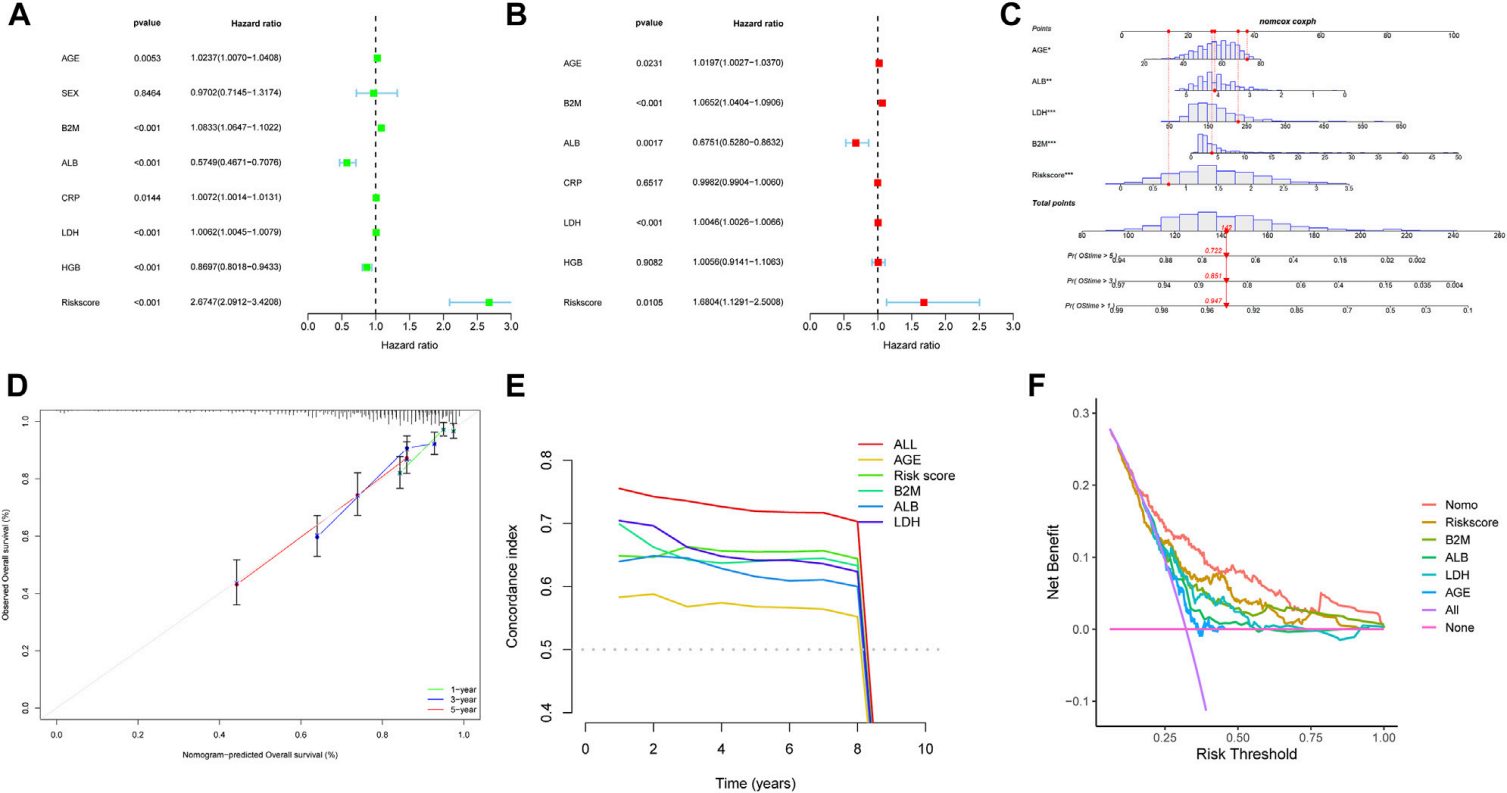
Nomogram construction based on the eight-gene signature and prognostic value of genes. Forest plot of univariate **(A)** and multivariate **(B)** Cox regression analysis of the clinical features in GSE24080. **(C)** Nomogram of risk score and other clinical factors for predicting MM1-, 3-, and 5-year overall survival in GSE24080. **(D)** The calibration plot **(D)**, C-index **(E)**, and DCA curves **(F)** of the nomogram in GSE24080.

We evaluated the correlation between the risk score and other clinical characteristics using univariate and multivariate Cox regression analysis with the GSE24080 dataset to determine whether the risk score was an independent prognostic factor. The univariate analysis revealed that AGE, β2M, ALB, CRP, LDH, HGB, and risk score were substantially associated with the overall survival in MM patients (Figure 6A). Then, multivariate analysis confirmed that AGE, β2M, ALB, LDH, and risk score were significant independent prognostic factors (Figure 6B). We established a nomogram using the four independent factors to predict 1-,3- and 5- year overall survival rate in the GSE24080 dataset (Figure 6C). We evaluated the nomogram in terms of calibration, discrimination, and DCA curve. The calibration curve demonstrated that the predictive performance of the nomogram was generally consistent with the actual survival time in terms of the 1-, 3- and 5-year overall survival rate (Figure 6D). The C-index (concordance index) is mainly used to evaluate the discrimination between predictive outcomes of the nomogram and actual outcomes in survival analysis. The nomogram’s C-index was at 0.727 (95% CI 0.706–0.749), showing that the nomogram has good discrimination (Figure 6E). The five-year DCA curves showed that the nomogram was a good predictor of survival in MM patients (Figure 6F).

### 3.5 Correlation of the risk model with clinical characteristics

**FIGURE 7:**
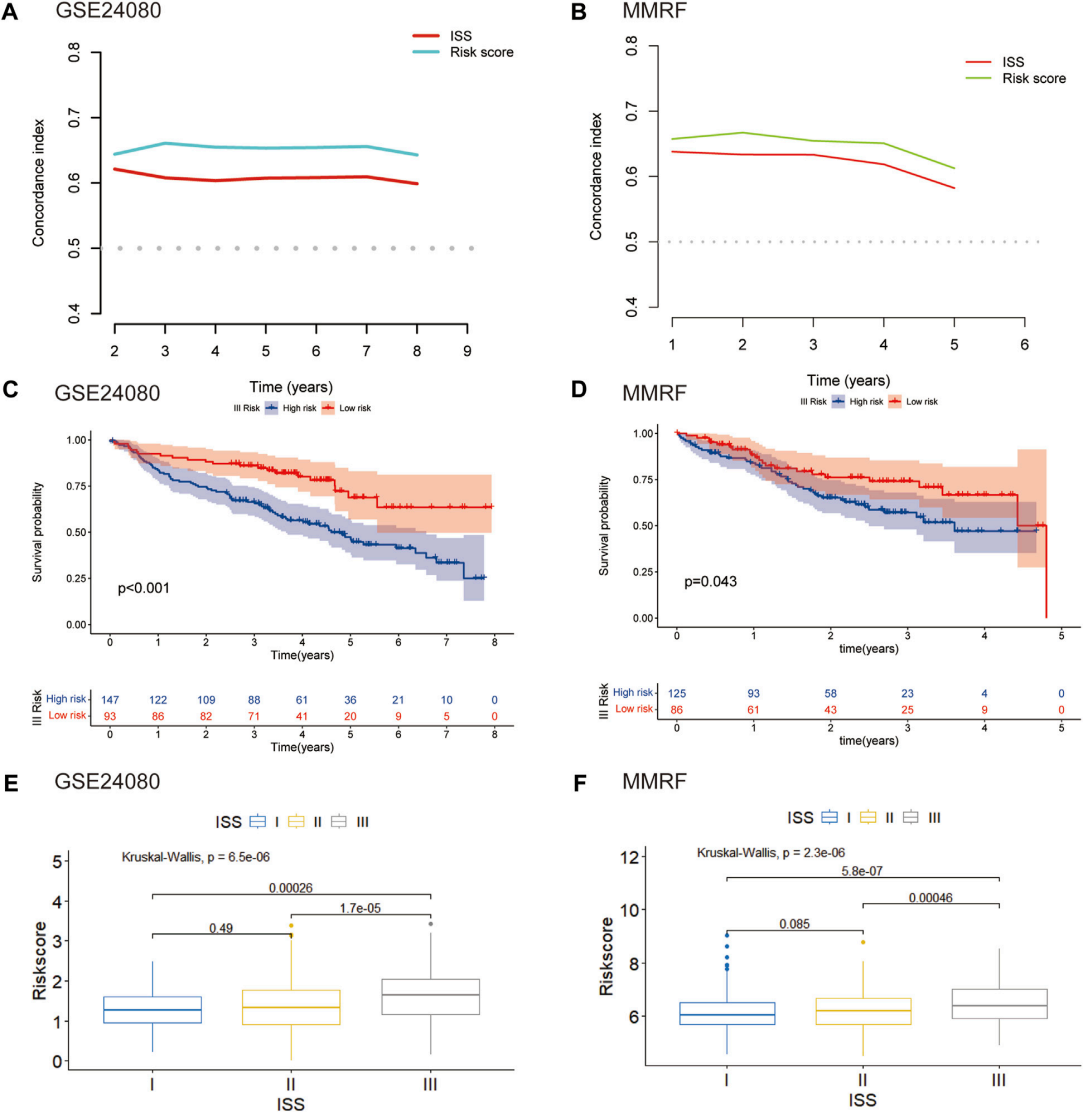
Validation of the eight-gene risk score model in ISS **(A)** The C-index of our risk model and ISS in GSE24080. **(B)** The C-index of our risk model and ISS in MMRF. **(C)** Kaplan–Meier curves of MM patients in stage III of ISS in GSE24080 (*p* < 0.001). **(D)** Kaplan–Meier curves of MM patients in stage III of ISS in MMRF cohort (*p* = 0.043). **(E)** Boxplot showed the difference in riskscore for different stages in GSE24080 (*p* < 0.001). **(F)** Boxplot showed the difference in risk scores for different stages in MMRF (*p* < 0001).

We further assessed the connection between the risk score and clinical features. The international staging system (ISS) is one of the earliest validated risk stratification for MM patients (Greipp et al., 2005). We first compared the predictive performance of this eight-gene risk model with ISS. In both GSE24080 and MMRF datasets, the time-dependent AUC of our model were higher than those of ISS, indicating that the predictive ability of our risk model was superior to ISS (Figures 7A, B). We then hypothesize that this risk model can further optimize the predictive performance of ISS for patient outcomes. We evaluated differences in overall survival between the high- and low-risk groups of three stages stratified by ISS. For the ISS stage I, the difference between the two groups was insignificant in both GSE24080 and MMRF datasets (Supplementary Figures S1A, S1B). For the ISS stage II, patients in the high-risk group had a worse prognosis than patients in the low-risk group in GSE24080. However, the difference between the two groups was not apparent in MMRF (Supplementary Figure S1C, S1D). As shown in Figures 7C, D, stage III patients were clearly split into two groups with varying survival rates, and the high-risk group had a worse prognosis. As shown in Figures 7E, F, the risk scores of GSE24080 and MMRF also increased significantly with increasing tumor stage (Kruskal–Wallis test *p* < 0.05, Figures 7E, F). When stage III patients were compared with stage I and II patients, respectively, there were significant differences in risk scores (Wilcoxon test *p* < 0.05). However, the difference in risk scores among stage I and stage II patients was not significant (Wilcoxon test *p* = 0.49, Wilcoxon test *p* = 0.089, respectively, Figures 7E, F). In conclusion, the eight-gene risk score model can be further used for stage III patients to predict the prognostic outcome more accurately.

**FIGURE 8:**
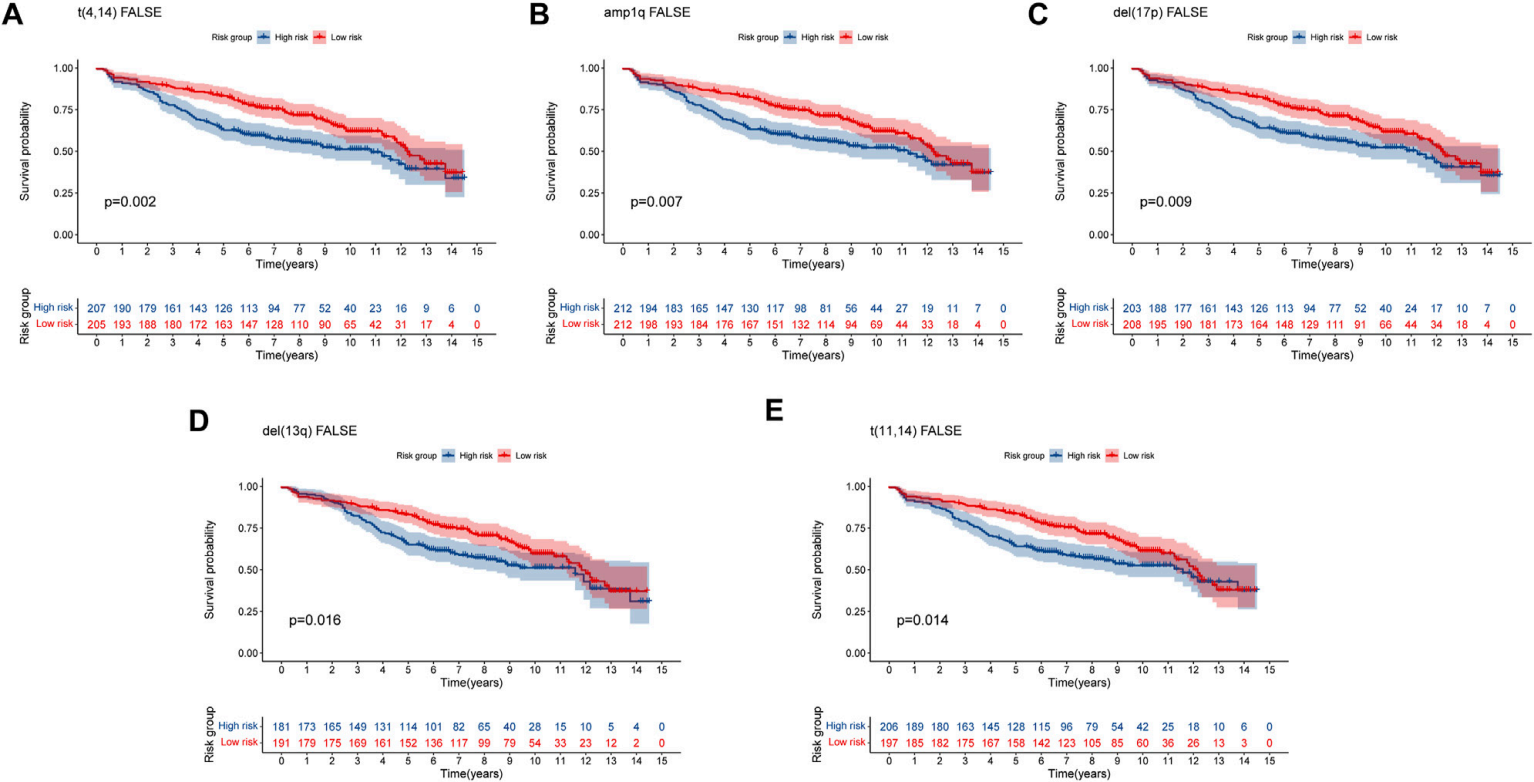
Validation of the eight-gene risk score model in patients without genetic indicators by Kaplan–Meier curves. **(A)** MM patients without t (Palumbo and Anderson, 2011; Lipchick et al., 2016) (*p* = 0.002). **(B)** MM patients without amp1q (*p* = 0.007). **(C)** MM patients without del (17p) (*p* = 0.009). **(D)** MM patients without del (13q) (*p* = 0.016) **(E)** MM patients without t (Schieber and Chandel, 2014; Lipchick et al., 2016) (*p* = 0.014). MM patients were divided into high-risk and low-risk groups by the median risk score.

Multiple myeloma (MM) is an exceptionally complicated and heterogeneous disease, which is characterized by various genetic alterations (Bustoros et al., 2022). Del (17p), del (13q) and translocation t (Palumbo and Anderson, 2011; Lipchick et al., 2016) were considered high-risk Chromosomal abnormalities by the IMWG (Palumbo et al., 2015). Amplification of 1q (amp1q) are associated with worse prognosis (D'Agostino et al., 2020). The prognostic significance of t (Schieber and Chandel, 2014; Lipchick et al., 2016) MM remains debatable (Bal et al., 2022). To confirm the predictive capacity of the 8-gene signature in patients with and without these genetic alterations, we selected the GSE136337 dataset containing these alterations for analysis. For genetic variation of amp1q, we only analyzed patients without amp1q, as there were only two patients with amp1q. We compared the survival of MM patients without these genetic indicators and found that patients in high-risk group had a worse prognosis (Figure 8). For patients with genetic indicators, including del (17p), del (13q), t (Palumbo and Anderson, 2011; Lipchick et al., 2016), and t (Schieber and Chandel, 2014; Lipchick et al., 2016), there was no difference between the high-risk group and low-risk group (Supplementary Figure S2). As a result, the MM patients without genetic abnormalities could be efficiently stratified by high- and low-risk using our eight-gene risk model.

### 3.6 Immune microenvironment of high- and low-risk groups

**FIGURE 9:**
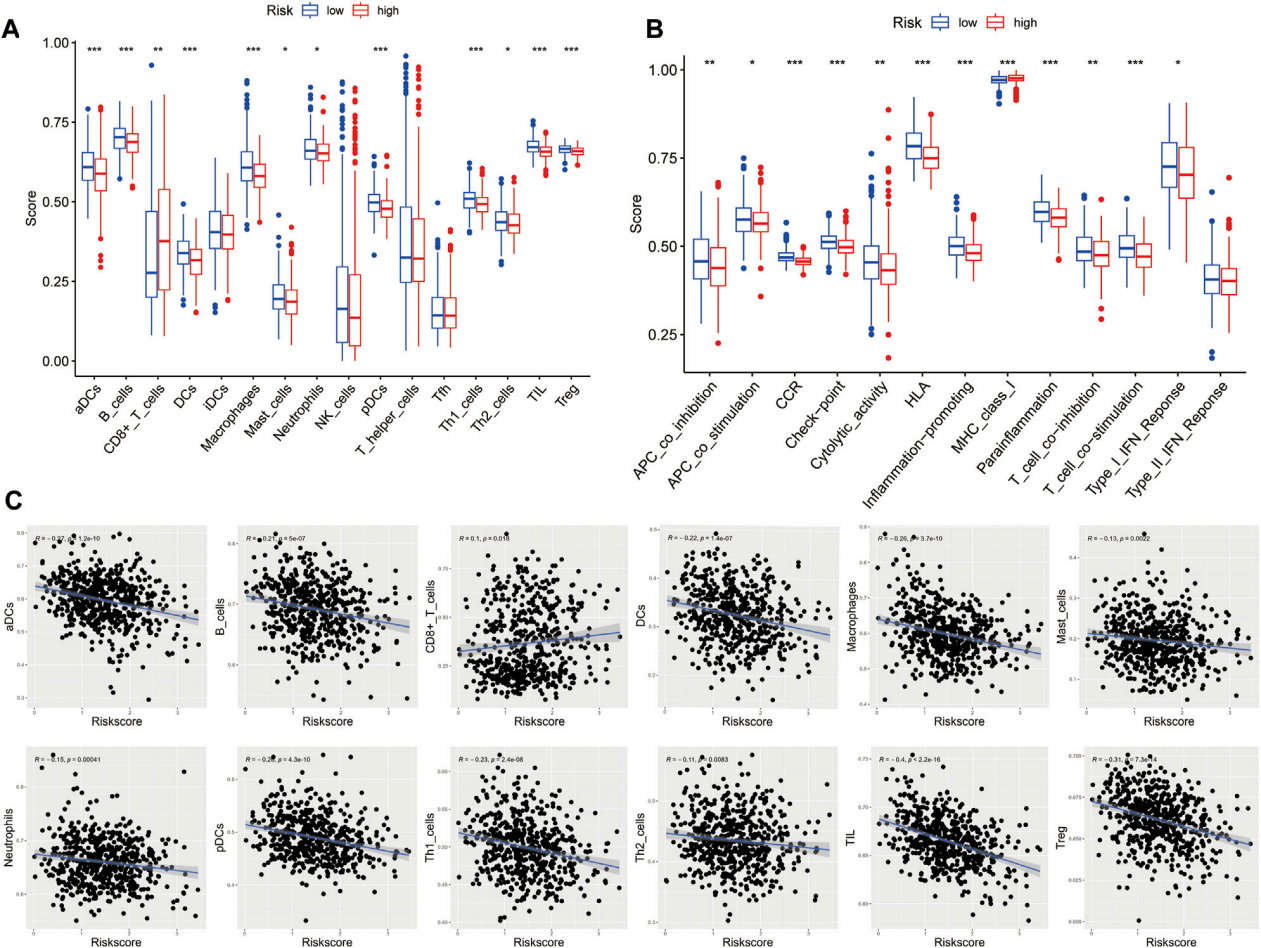
Characteristics of immune cell and immune function in different risk groups. **(A)** Comparison of the 16 immune cell scores calculated by ssGSEA between different risk groups.**(B)** Comparison of the 13 immune-related functions by ssGSEA between different risk groups. **(C)** Relations between immune cells and the risk score (*p < 0.05; **p < 0.01; ***p < 0.001)

To further investigate the relationship between risk score and immune infiltration, we used ssGSEA to evaluate the enrichment scores of different immune cells and immunological functions in the high-risk and low-risk groups. Compared to the high-risk group, most immune cell components were higher in the low-risk group (such as aDCs, macrophages, pDCs, Th1 cells, DCs, B cells, mast cells, Th2 cells, T helper cells, TIL, Treg), except for a lower proportion of CD8^+^ T cells. Moreover, except for MHC class I, the immune functional scores, such as APC co-inhibition, T cell co-inhibition, neutrophils, Type I IFN response, CCR, cytolytic activity, APC co-stimulation, HLA, T cell co-stimulation, inflammation-promoting, checkpoint, and parainflammation were lower in the high-risk group than in the low-risk group (Figures 9A, B). We further analyzed the correlation between the enrichment scores for immune cell and risk scores. The results showed a negative correlation between the risk score and the enrichment scores of aDCs, macrophages, pDCs, Th1 cells, DCs, B cells, mast cells, Th2 cells, T helper cells, TIL, and Treg, while the risk score and the enrichment score of CD8^+^ T cells were positively correlated (Figure 9C). These findings may suggest that the immune system of low-risk patients is more active.

### 3.7 Chemotherapy sensitivity prediction

**FIGURE 10:**
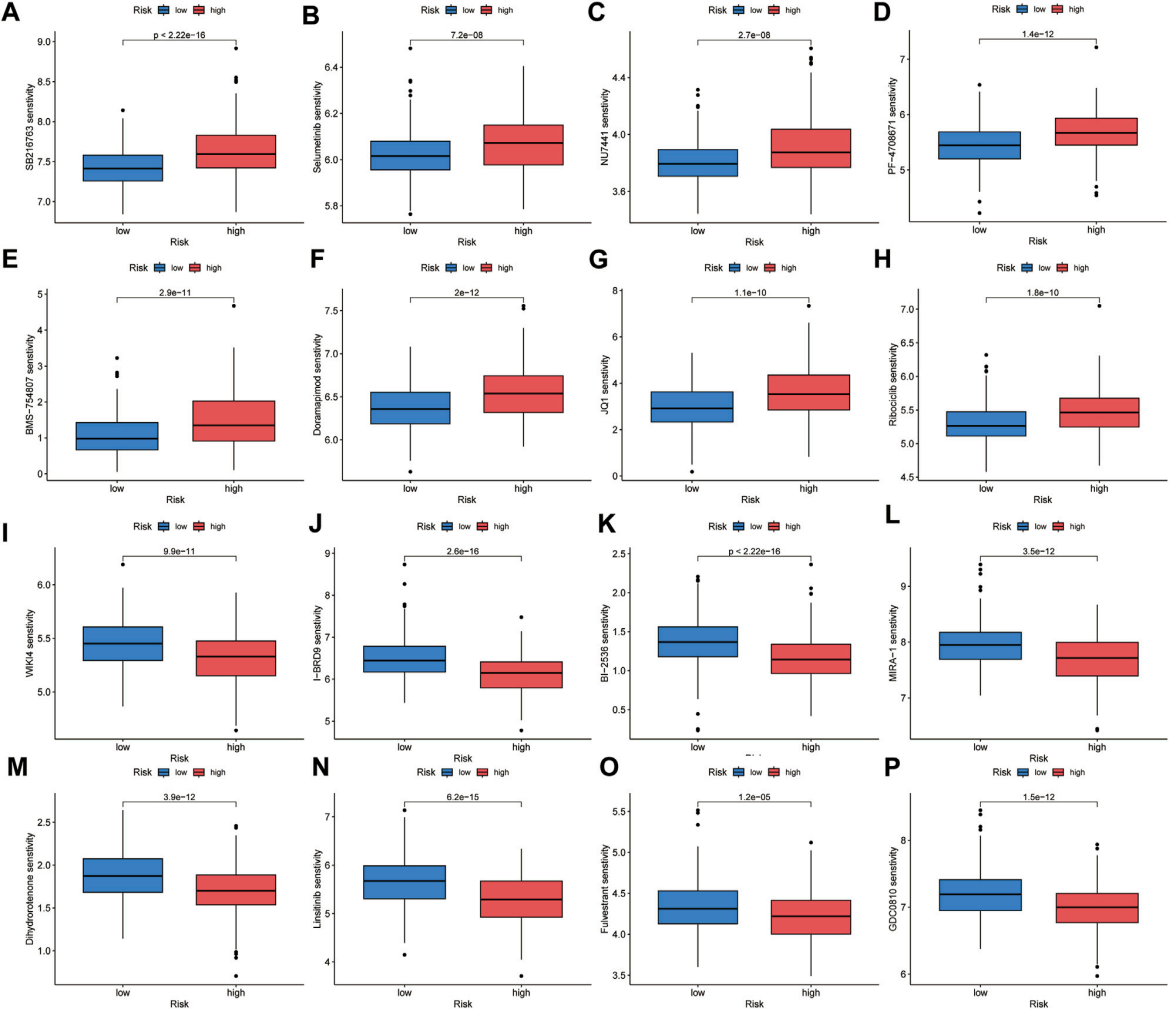
Chemotherapeutic sensitivity prediction. High-risk group showed lower sensitivity to SB216763 **(A)**, Selumetinib **(B)**, NU7441 **(C)**, PF-4708671 **(D)**, BMS-754807 **(E)**, Doramapimod **(F)**, JQ1 **(G)** and Ribociclib **(H)**, and higher sensitivity to WIKI4 **(I)**, I-BRD9 **(J)**, BI-2536 **(K)**, MIRA-1 **(L)**, Dihydrorotenone **(M)**, Linsitinib **(N)**, Fulvestrant **(O)** and GDC0810 **(P)**.

To identify suitable drugs for high-risk patients, we compared the chemosensitivity of high- and low-risk groups based on IC50 values using the OncoPredict algorithm. A lower IC50 value indicates higher drug sensitivity. We found that patients in the high-risk group exhibited greater resistance to eight drugs, including SB216763 (a GSK3 inhibitor), Doramapimod (a p38 MAPK inhibitor), PF-4708671 (an S6K1 inhibitor), BMS-754807 (an IGF-1R/IR inhibitor), Selumetinib (a MEK inhibitor), NU7441 (a DNAPK inhibitor), Ribociclib (a CDK4/CDK6 inhibitor), and JQ1 (a BET inhibitor). On the other hand, high-risk patients showed greater sensitivity to eight drugs, including MIRA-1 (a TP53 inhibitor), GDC0810 (an ESR1/ESR2 inhibitor), Dihydrorotenone (a mitochondrial inhibitor), I-BRD9 (a BRD9 inhibitor), WIKI4 (a TNKS1/TNKS2 inhibitor), Fulvestrant (an ESR inhibitor), Linsitinib (an IGF1R inhibitor), and BI-2536 (a PLK1/PLK2/PLK3 inhibitor) (Figure 10). We believe that these drugs may be beneficial for the subsequent treatment of high-risk patients.

## 4 Discussion

Multiple myeloma is a highly heterogeneous hematologic malignancy, and the clinical outcomes of MM patients vary widely. What they have in common is that most cases are incurable (Siegel et al., 2019). Cell death is essential for maintaining of organismal homeostasis, and preventing excessive proliferation. Cuproptosis is a newly discovered form of death, which is closely associated with mitochondrial metabolic activity (Tsvetkov et al., 2022). Oxidative stress plays a critical role in various stages of tumorigenesis and cancer progression. Excessive ROS causes mitochondrial DNA damage, which associated with initiates tumorigenesis (Cadenas and Davies, 2000). Both cuprotosis and oxidative stress are closely related to mitochondrial homeostasis. Oxidative stress also plays an indispensable role in cell death, which can result in ferroptosis and autophagy (Chen et al., 2022; Ma et al., 2022). We combined the two in our analysis.

In our study, we first identified cuproptosis-related oxidative stress genes and then obtained DEGs between healthy donors and MM patients, after which the two were taken to intersect to obtain 76 DECROGs. A novel 8 gene signature as a prognostic marker for MM was developed by univariate, LASSO, and multivariable Cox analysis. Patients were divided into the high- and low-risk groups according to the median cut-point of risk score. There are significant differences in the Kaplan–Meier survival curve between the high-risk and low-risk groups in GSE24080. Similar results were observed in other three GEO and MMRF cohorts.

The eight genes in the risk score model: *CCNB1* (Cyclin B1) is associated with the cell cycle and mitosis. In previous study, comparing to replased MM patient, the level of *CCNB1* was downregulated in newly diagnosed MM patients (Dementyeva et al., 2013). *MIF* (Macrophage Migration Inhibitory Factor) has been shown to accelerates the development of the disease in multiple myeloma. MM patients with high *MIF* had a worse prognosis (Wang et al., 2020; Xu et al., 2021). A number of studies have shown that *PLA2G4A* (placental phospholipase A24A is involved in the biological procedure of stress response in various diseases (Brien et al., 2017; Zhang et al., 2018; Mishra et al., 2022). *PLA2G4A* is overexpressed in different solid cancers such as hepatocellular carcinoma (Fu et al., 2017), prostate cancer (Patel et al., 2008), breast cancer (Chen et al., 2017), cervical cancer (Xu et al., 2019), as well as malignant hematologic diseases, such as AML (acute myeloid leukemia) (Runarsson et al., 2007; Bai et al., 2020; Zhang et al., 2022) In multiple myeloma patients, expression of the *PLA2G4A* was higher compared to healthy individuals and high gene expression of *PLA2G4A* is associated with adverse outcomes (Mahammad et al., 2021). A previous study showed that *FOXO1* (Forkhead Box O1) activation could inhibit the tumor growth and induce cell autophagy and cell death. In Chronic myeloid leukemia, FOXO1 is upregulated in drug-resistant cells with BCR-ABL1 kinase mutation (Wagle et al., 2016). In MM, FOXO1 mediates cell apoptosis and inhibits tumor cell growth (Liu et al., 2016). *RNASE3* (Ribonuclease A Family Member 3), also known as *ECP*(Eosinophil cationic protein), is mainly involved in human immune function (Ostendorf et al., 2020). A previous study described the protective role of *ECP* under oxidative stress. It inhibits ROS-induced apoptosis in cardiomyocytes *via PI3K-Akt* pathway (Ishii et al., 2015). In hematological cancers, it was significantly upregulated in CML patients’ PBMCs compared with healthy controls (Yao et al., 2022). *APOE* (apolipoprotein E) is a known immune suppressant, which is significantly regulated in many human tumors, but its role in multiple myeloma has not been defined (Wu et al., 2022). *KIT* (KIT proto-oncogene) encodes a receptor tyrosine kinase. Its role in MM needs to be further investigated. *EGR1* (early growth response protein 1) is characterized as a tumor suppressor in multiple myeloma (Chen et al., 2010).

A nomogram integrates multiple independent prognostic factors, and scores of each factor are calculated based on their contribution to the outcome. Then a total score for an individual patient can be calculated. Finally, the outcome for a given patient can be predictive. Therefore, we can specify a more precise treatment strategy based on the results of the nomogram (Iasonos et al., 2008). In this study, we evaluated the relations between DECROGs and patients’ clinical characteristics, and multivariate Cox regression analysis showed that risk score was one of the independent prognostic factors indicating that the DECROGs could serve as a reliable predictor of survival. Then we constructed a nomogram to predict the outcome of MM patients. The C-index, calibration curve and DCA curve demonstrated that the nomogram’s predicted value was in close agreement with the actual outcome.

The International Staging System (ISS) is the standard for staging of myeloma. Therefore, we wanted to evaluate whether the model could improve prognosis prediction in MM patients by combining with the ISS. The outcomes demonstrated that the model could optimize ISS to some extent by further differentiating patients with ISS stage III. Chromosomal change are present in the plasma cell of almost 100% of MM patients (Avet-Loiseau et al., 2009), which resulting the internal heterogeneity of MM. We evaluate the role of our model in patients with or without genetic alterations. In patients with genetic alterations, including del (17q), t (Palumbo and Anderson, 2011; Lipchick et al., 2016), del (13q), amp1q, and t (Chu et al., 2012; Schieber and Chandel, 2014), the difference was not significant, whereas in patients without these alterations, our model allows further stratification of patients by prognosis. The high-risk group demonstrated shorter overall survival than the low-risk group. Above all, the model can increase the prediction accuracy significantly combining ISS and genetic alterations.

As immune cells play a crucial role in the development of multiple myeloma, we further analyzed the relationship between the model and immune infiltration (García-Ortiz et al., 2021). In multiple myeloma, cell-mediated immunity is suppressed (Kyle and Rajkumar, 2004). Our study found that compared to the low-risk group, most immune cells were lacking in the high-risk group, indicating an overall decrease in immune activity. The reduction of these immune cells may lead to increased susceptibility to infection and the development of other diseases, and may also affect the patient’s response to multiple myeloma treatment, which is unfavorable for prognosis (Allegra et al., 2021).

Drug sensitivity research is beneficial for discovering potential therapeutic drugs. We used the Oncopredict tool to evaluate the IC50 of different drugs in the high-risk and low-risk groups by analyzing cancer drug sensitivity genomics (GDSC). High-risk patients were found to be more sensitive to MIRA-1, GDC0810, Dihydrorotenone, I-BRD9, WIKI4, Fulvestrant.1, Linsitinib, and BI-2536, providing candidate drugs for the treatment of multiple myeloma.

There are inevitably some limitations in the research work, which we hope to solve in the future.

First, this is a retrospective study and needs further prospective studies to confirm our results. Second, to increase the sample size, we employed numerous datasets. Biases between platforms are unavoidable, which may cause differences in the results. Moreover, the role of the eight-gene signature in the pathogenesis of MM remains to be addressed based on further experimental research.

In conclusion, we established an eight-gene risk model based on oxidative stress genes associated with cuproptosis. The risk model and prognosis are significantly correlated. Combining the risk model with clinical features, we established a novel nomogram that may better predict the survival in MM patients more accurately. This study offers a novel perspective on understanding multiple myeloma and provides potential targets for the diagnosis and treatment of multiple myeloma.

## Data Availability

Publicly available datasets were analyzed in this study. This data can be found here: https://www.ncbi.nlm.nih.gov/geo/ https://research.themmrf.org/.

## References

[B1] Allegra A. Tonacci A. Musolino C. Pioggia G. Gangemi S. (2021). Secondary immunodeficiency in hematological malignancies: Focus on multiple myeloma and chronic lymphocytic leukemia. Front. Immunol. 12, 738915. 10.3389/fimmu.2021.738915 34759921 PMC8573331

[B2] Avet-Loiseau H. Li C. Magrangeas F. Gouraud W. Charbonnel C. Harousseau J-L. (2009). Prognostic significance of copy-number alterations in multiple myeloma. J. Clin. Oncol. 27 (27), 4585–4590. 10.1200/JCO.2008.20.6136 19687334 PMC2754906

[B3] Bai H. Zhou M. Zeng M. Han L. (2020). *PLA2G4A* is a potential biomarker predicting shorter overall survival in patients with non-M3/*NPM1* wildtype acute myeloid leukemia. DNA Cell Biol. 39 (4), 700–708. 10.1089/dna.2019.5187 32077754

[B4] Bal S. Kumar S. K. Fonseca R. Gay F. Hungria V. T. Dogan A. (2022). Multiple myeloma with t(11;14): Unique biology and evolving landscape. Am. J. cancer Res. 12 (7), 2950–2965. 35968339 PMC9360221

[B5] Brien M. Larose J. Greffard K. Julien P. Bilodeau J. F. (2017). Increased placental phospholipase A_2_ gene expression and free F_2_-isoprostane levels in response to oxidative stress in preeclampsia. Placenta 55, 54–62. 10.1016/j.placenta.2017.05.004 28623974

[B6] Bustoros M. Anand S. Sklavenitis-Pistofidis R. Redd R. Boyle E. M. Zhitomirsky B. (2022). Genetic subtypes of smoldering multiple myeloma are associated with distinct pathogenic phenotypes and clinical outcomes. Nat. Commun. 13 (1), 3449. 10.1038/s41467-022-30694-w 35705541 PMC9200804

[B7] Cadenas E. Davies K. J. (2000). Mitochondrial free radical generation, oxidative stress, and aging. Free Radic. Biol. Med. 29 (3-4), 222–230. 10.1016/s0891-5849(00)00317-8 11035250

[B8] Chen K. Ma S. Deng J. Jiang X. Ma F. Li Z. (2022). Ferroptosis: A new development trend in periodontitis. Cells 11 (21), 3349. 10.3390/cells11213349 36359745 PMC9654795

[B9] Chen L. Fu H. Luo Y. Chen L. Cheng R. Zhang N. (2017). cPLA2α mediates TGF-β-induced epithelial-mesenchymal transition in breast cancer through PI3k/Akt signaling. Cell Death Dis. 8 (4), e2728. 10.1038/cddis.2017.152 28383549 PMC5477578

[B10] Chen L. Wang S. Zhou Y. Wu X. Entin I. Epstein J. (2010). Identification of early growth response protein 1 (EGR-1) as a novel target for JUN-induced apoptosis in multiple myeloma. Blood 115 (1), 61–70. 10.1182/blood-2009-03-210526 19837979 PMC2803692

[B11] Chng W. J. Kumar S. Vanwier S. Ahmann G. Price-Troska T. Henderson K. (2007). Molecular dissection of hyperdiploid multiple myeloma by gene expression profiling. Cancer Res. 67 (7), 2982–2989. 10.1158/0008-5472.CAN-06-4046 17409404

[B12] Chu L. Su M. Y. Maggi L. B. Jr. Lu L. Mullins C. Crosby S. (2012). Multiple myeloma-associated chromosomal translocation activates orphan snoRNA ACA11 to suppress oxidative stress. J. Clin. investigation 122 (8), 2793–2806. 10.1172/JCI63051 PMC3408744 22751105

[B13] D'Agostino M. Ruggeri M. Aquino S. Giuliani N. Arigoni M. Gentile M. (2020). Impact of gain and amplification of 1q in newly diagnosed multiple myeloma patients receiving carfilzomib-based treatment in the forte trial. Blood 136, 38–40. 10.1182/blood-2020-137060

[B14] Danziger S. A. McConnell M. Gockley J. Young M. H. Rosenthal A. Schmitz F. (2020). Bone marrow microenvironments that contribute to patient outcomes in newly diagnosed multiple myeloma: A cohort study of patients in the total therapy clinical trials. PLoS Med. 17 (11), e1003323. 10.1371/journal.pmed.1003323 33147277 PMC7641353

[B15] Dementyeva E. Kryukov F. Kubiczkova L. Nemec P. Sevcikova S. Ihnatova I. (2013). Clinical implication of centrosome amplification and expression of centrosomal functional genes in multiple myeloma. J. Transl. Med. 11, 77. 10.1186/1479-5876-11-77 23522059 PMC3615957

[B16] Friedman J. Hastie T. Tibshirani R. (2010). Regularization paths for generalized linear models via coordinate descent. J. Stat. Softw. 33 (1), 1–22. 10.18637/jss.v033.i01 20808728 PMC2929880

[B17] Fu H. He Y. Qi L. Chen L. Luo Y. Chen L. (2017). cPLA2α activates PI3K/AKT and inhibits Smad2/3 during epithelial-mesenchymal transition of hepatocellular carcinoma cells. Cancer Lett. 403, 260–270. 10.1016/j.canlet.2017.06.022 28649002

[B18] García-Ortiz A. Rodríguez-García Y. Encinas J. Maroto-Martín E. Castellano E. Teixidó J. (2021). The role of tumor microenvironment in multiple myeloma development and progression. Cancers 13 (2), 217. 10.3390/cancers13020217 33435306 PMC7827690

[B19] Greipp P. R. San Miguel J. Durie B. G. M. Crowley J. J. Barlogie B. Bladé J. (2005). International staging system for multiple myeloma. J. Clin. Oncol. 23 (15), 3412–3420. 10.1200/JCO.2005.04.242 15809451

[B20] Hanamura I. Huang Y. Zhan F. Barlogie B. Shaughnessy J. (2006). Prognostic value of cyclin D2 mRNA expression in newly diagnosed multiple myeloma treated with high-dose chemotherapy and tandem autologous stem cell transplantations. Leukemia 20 (7), 1288–1290. 10.1038/sj.leu.2404253 16688228

[B21] Hänzelmann S. Castelo R. Guinney J. (2013). Gsva: Gene set variation analysis for microarray and RNA-seq data. BMC Bioinforma. 14, 7. 10.1186/1471-2105-14-7 PMC3618321 23323831

[B22] Holmström K. M. Finkel T. (2014). Cellular mechanisms and physiological consequences of redox-dependent signalling. Nat. Rev. Mol. Cell Biol. 15 (6), 411–421. 10.1038/nrm3801 24854789

[B23] Iasonos A. Schrag D. Raj G. V. Panageas K. S. (2008). How to build and interpret a nomogram for cancer prognosis. J. Clin. Oncol. 26 (8), 1364–1370. 10.1200/JCO.2007.12.9791 18323559

[B24] Ishii H. Kamikawa S. Hirohata S. Mizutani A. Abe K. Seno M. (2015). Eosinophil cationic protein shows survival effect on H9c2 cardiac myoblast cells with enhanced phosphorylation of ERK and akt/GSK-3β under oxidative stress. Acta Med. Okayama 69 (3), 145–153. 10.18926/AMO/53521 26101190

[B25] Joshua D. E. Bryant C. Dix C. Gibson J. Ho J. (2019). Biology and therapy of multiple myeloma. Med. J. Aust. 210 (8), 375–380. 10.5694/mja2.50129 31012120

[B26] Kim B. E. Nevitt T. Thiele D. J. (2008). Mechanisms for copper acquisition, distribution and regulation. Nat. Chem. Biol. 4 (3), 176–185. 10.1038/nchembio.72 18277979

[B27] Kyle R. A. Rajkumar S. V. (2004). Multiple myeloma. N. Engl. J. Med. 351 (18), 1860–1873. 10.1056/NEJMra041875 15509819

[B28] Lipchick B. C. Fink E. E. Nikiforov M. A. (2016). Oxidative stress and proteasome inhibitors in multiple myeloma. Pharmacol. Res. 105, 210–215. 10.1016/j.phrs.2016.01.029 26827824 PMC5044866

[B29] Liu X. Zhang Y. Wang Z. Wang X. Zhu G. Han G. (2016). Metabotropic glutamate receptor 3 is involved in B-cell-related tumor apoptosis. Int. J. Oncol. 49 (4), 1469–1478. 10.3892/ijo.2016.3623 27431857

[B30] Ma Y. Su Q. Yue C. Zou H. Zhu J. Zhao H. (2022). The effect of oxidative stress-induced autophagy by cadmium exposure in kidney, liver, and bone damage, and neurotoxicity. Int. J. Mol. Sci. 23 (21), 13491. 10.3390/ijms232113491 36362277 PMC9659299

[B31] Maeser D. Gruener R. F. Huang R. S. (2021). oncoPredict: an R package for predicting *in vivo* or cancer patient drug response and biomarkers from cell line screening data. Briefings Bioinforma. 22 (6), bbab260. 10.1093/bib/bbab260 PMC8574972 34260682

[B32] Mahammad N. Ashcroft F. J. Feuerherm A. J. Elsaadi S. Vandsemb E. N. Børset M. (2021). Inhibition of cytosolic phospholipase A2α induces apoptosis in multiple myeloma cells. Mol. (Basel, Switz. 26 (24), 7447. 10.3390/molecules26247447 PMC8705991 34946532

[B33] Mishra S. Charan M. Shukla R. K. Agarwal P. Misri S. Verma A. K. (2022). cPLA2 blockade attenuates S100A7-mediated breast tumorigenicity by inhibiting the immunosuppressive tumor microenvironment. J. Exp. Clin. cancer Res. CR 41 (1), 54. 10.1186/s13046-021-02221-0 35135586 PMC8822829

[B34] Mitsiades C. S. Mitsiades N. S. Richardson P. G. Munshi N. C. Anderson K. C. (2007). Multiple myeloma: A prototypic disease model for the characterization and therapeutic targeting of interactions between tumor cells and their local microenvironment. J. Cell. Biochem. 101 (4), 950–968. 10.1002/jcb.21213 17546631

[B35] Oliveri V. (2022). Selective targeting of cancer cells by copper ionophores: An overview. Front. Mol. Biosci. 9, 841814. 10.3389/fmolb.2022.841814 35309510 PMC8931543

[B36] Ostendorf T. Zillinger T. Andryka K. Schlee-Guimaraes T. M. Schmitz S. Marx S. (2020). Immune sensing of synthetic, bacterial, and Protozoan RNA by toll-like receptor 8 requires coordinated processing by RNase T2 and RNase 2. Immunity 52 (4), 591–605.e6. 10.1016/j.immuni.2020.03.009 32294405

[B37] Palumbo A. Anderson K. (2011). Multiple myeloma. N. Engl. J. Med. 364 (11), 1046–1060. 10.1056/NEJMra1011442 21410373

[B38] Palumbo A. Avet-Loiseau H. Oliva S. Lokhorst H. M. Goldschmidt H. Rosinol L. (2015). Revised international staging system for multiple myeloma: A report from international myeloma working group. J. Clin. Oncol. 33 (26), 2863–2869. 10.1200/JCO.2015.61.2267 26240224 PMC4846284

[B39] Patel M. I. Singh J. Niknami M. Kurek C. Yao M. Lu S. (2008). Cytosolic phospholipase A2-alpha: A potential therapeutic target for prostate cancer. Clin. cancer Res. 14 (24), 8070–8079. 10.1158/1078-0432.CCR-08-0566 19088022 PMC2605658

[B40] Rae T. D. Schmidt P. J. Pufahl R. A. Culotta V. C. O'Halloran T. V. (1999). Undetectable intracellular free copper: The requirement of a copper chaperone for superoxide dismutase. Science 284 (5415), 805–808. 10.1126/science.284.5415.805 10221913

[B41] Rajkumar S. V. Dimopoulos M. A. Palumbo A. Blade J. Merlini G. Mateos M-V. (2014). International Myeloma Working Group updated criteria for the diagnosis of multiple myeloma. Lancet Oncol. 15 (12), e538–e548. 10.1016/S1470-2045(14)70442-5 25439696

[B42] Runarsson G. Feltenmark S. Forsell P. K. A. Sjöberg J. Björkholm M. Claesson H-E. (2007). The expression of cytosolic phospholipase A2 and biosynthesis of leukotriene B4 in acute myeloid leukemia cells. Eur. J. Haematol. 79 (6), 468–476. 10.1111/j.1600-0609.2007.00967.x 17976189

[B43] Schieber M. Chandel N. S. (2014). ROS function in redox signaling and oxidative stress. Curr. Biol. 24 (10), R453–R462. 10.1016/j.cub.2014.03.034 24845678 PMC4055301

[B44] Shi L. Campbell G. Jones W. D. Campagne F. Wen Z. Walker S. J. (2010). The MicroArray Quality Control (MAQC)-II study of common practices for the development and validation of microarray-based predictive models. Nat. Biotechnol. 28 (8), 827–838. 10.1038/nbt.1665 20676074 PMC3315840

[B45] Siegel R. L. Miller K. D. Jemal A. (2019). Cancer statistics. CA a cancer J. Clin. 69, 7–34. 10.3322/caac.21551 30620402

[B46] Sonneveld P. Avet-Loiseau H. Lonial S. Usmani S. Siegel D. Anderson K. C. (2016). Treatment of multiple myeloma with high-risk cytogenetics: A consensus of the international myeloma working group. Blood 127 (24), 2955–2962. 10.1182/blood-2016-01-631200 27002115 PMC4920674

[B47] Tang D. Chen X. Kroemer G. (2022). Cuproptosis: A copper-triggered modality of mitochondrial cell death. Cell Res. 32 (5), 417–418. 10.1038/s41422-022-00653-7 35354936 PMC9061796

[B48] Tsvetkov P. Coy S. Petrova B. Dreishpoon M. Verma A. Abdusamad M. (2022). Copper induces cell death by targeting lipoylated TCA cycle proteins. Sci. (New York, NY) 375 (6586), 1254–1261. 10.1126/science.abf0529 PMC9273333 35298263

[B49] Wagle M. Eiring A. M. Wongchenko M. Lu S. Guan Y. Wang Y. (2016). A role for FOXO1 in BCR-ABL1-independent tyrosine kinase inhibitor resistance in chronic myeloid leukemia. Leukemia 30 (7), 1493–1501. 10.1038/leu.2016.51 27044711 PMC4935980

[B50] Wang Q. Zhao D. Xian M. Wang Z. Bi E. Su P. (2020). MIF as a biomarker and therapeutic target for overcoming resistance to proteasome inhibitors in human myeloma. Blood 136 (22), 2557–2573. 10.1182/blood.2020005795 32582913 PMC7714094

[B51] Wu X. Srinivasan P. Basu M. Zhang P. Saruwatari M. Thommandru B. (2022). Tumor Apolipoprotein E is a key checkpoint blocking anti-tumor immunity in mouse melanoma. Front. Immunol. 13, 991790. 10.3389/fimmu.2022.991790 36341364 PMC9626815

[B52] Wu Z. Wang L. Wen Z. Yao J. (2021). Integrated analysis identifies oxidative stress genes associated with progression and prognosis in gastric cancer. Sci. Rep. 11 (1), 3292. 10.1038/s41598-021-82976-w 33558567 PMC7870842

[B53] Xu H. Sun Y. Zeng L. Li Y. Hu S. He S. (2019). Inhibition of cytosolic phospholipase A2 alpha increases chemosensitivity in cervical carcinoma through suppressing β-catenin signaling. Cancer Biol. Ther. 20 (6), 912–921. 10.1080/15384047.2019.1579961 30829552 PMC6605985

[B54] Xu J. Yu N. Zhao P. Wang F. Huang J. Cui Y. (2021). Intratumor heterogeneity of MIF expression correlates with extramedullary involvement of multiple myeloma. Front. Oncol. 11, 694331. 10.3389/fonc.2021.694331 34268123 PMC8276700

[B55] Yang W. Soares J. Greninger P. Edelman E. J. Lightfoot H. Forbes S. (2013). Genomics of drug sensitivity in cancer (GDSC): A resource for therapeutic biomarker discovery in cancer cells. Nucleic acids Res. 41, D955–D961. 10.1093/nar/gks1111 23180760 PMC3531057

[B56] Yao F. Zhao C. Zhong F. Qin T. Li S. Liu J. (2022). Bioinformatics analysis and identification of hub genes and immune-related molecular mechanisms in chronic myeloid leukemia. PeerJ 10, e12616. 10.7717/peerj.12616 35111390 PMC8781323

[B57] Yruela I. (2009). Copper in plants: Acquisition, transport and interactions. Funct. plant Biol. FPB 36 (5), 409–430. 10.1071/FP08288 32688656

[B58] Yu G. Wang L. G. Han Y. He Q. Y. (2012). clusterProfiler: an R package for comparing biological themes among gene clusters. Omics a J. Integr. Biol. 16 (5), 284–287. 10.1089/omi.2011.0118 PMC3339379 22455463

[B59] Zhang J. Chen Z. Wang F. Xi Y. Hu Y. Guo J. (2022). Machine learning assistants construct oxidative stress-related gene signature and discover potential therapy targets for acute myeloid leukemia. Oxidative Med. Cell. Longev. 2022, 1507690. 10.1155/2022/1507690 PMC9423988 36046688

[B60] Zhang W. Wang X. Zhang L. Geng D. Wang Y. Sun D. (2018). Inhibition of PLA2G4A reduces the expression of lung cancer-related cytokines. DNA Cell Biol. 2018. 10.1089/dna.2018.4286 30328712

