# Supplementary material for: Comprehensive analysis of prognosis of cuproptosis-related oxidative stress genes in multiple myeloma
Source: Front Genet. 2023 Mar 31;14:1100170. doi: 10.3389/fgene.2023.1100170 (PMC10102368; doi:10.3389/fgene.2023.1100170)
Supplement: Supplementary file 2 [file DataSheet2.ZIP › Supplementary_Material.docx]

Supplementary Material

## Supplementary Figures

**Supplement Table 1. Cuproptosis-Related Genes**

**Supplement Table 2. Oxidative Stress-Related Genes**

**Supplement Figure 1. Validation of the eight-gene risk score model in ISS** (A) Kaplan–Meier curves of MM patients in stage I in GSE24080 (p = 0.999). (B) Kaplan–Meier curves of MM patients in stage I and stage II in MMRF cohort (p = 0.208) (C) Kaplan–Meier curves of MM patients in stage II in GSE24080 (p<0.001). (D) Kaplan–Meier curves of MM patients in stage II in MMRF cohort (p=0.121)

**Supplement Figure 2.Validation of the eight-gene risk score model in patients with genetic indicators by Kaplan–Meier curves.** (A) MM patients without with (4,14)(p = 0.227). (B) MM patients with del(13q) (p = 0.105). (C) MM patients without del (17p) (p = 0.347). (D) MM patients without t(11;14) ( p = 0.240). MM patients were divided into high-risk and low-risk groups by the median risk score.
