# Supplementary material for: Comprehensive analysis of prognosis of cuproptosis-related oxidative stress genes in multiple myeloma
Source: Front Genet. 2023 Mar 31;14:1100170. doi: 10.3389/fgene.2023.1100170 (PMC10102368; doi:10.3389/fgene.2023.1100170)
Supplement: Supplementary file 2 [file DataSheet2.ZIP › Supplement Table1.docx]

**Supplement Table1 Cuproptosis-Related Genes**

| FDX1 |
| --- |
| LIPT1 |
| LIAS |
| DLD |
| DBT |
| GCSH |
| DLST |
| DLAT |
| PDHA1 |
| PDHB |
| SLC31A1 |
| ATP7A |
| ATP7B |
