# Supplementary material for: Comprehensive analysis of prognosis of cuproptosis-related oxidative stress genes in multiple myeloma
Source: Front Genet. 2023 Mar 31;14:1100170. doi: 10.3389/fgene.2023.1100170 (PMC10102368; doi:10.3389/fgene.2023.1100170)
Supplement: Supplementary file 2 [file DataSheet2.ZIP › Supplement Table2.docx]

**Supplement Table2. Oxidative Stress-Related Genes**

| KIT | NDUFA13 | MAPK13 | TNFSF4 | LEPQTL1 |
| --- | --- | --- | --- | --- |
| MIR185 | ALS3 | ALS7 | OXTR | DHFR |
| ACACA | GPX8 | BGLAP | TAZ | MAPK12 |
| YBX1 | SFXN4 | SLC4A1 | HPSE | RPTOR |
| MTA1 | CD274 | ENDOG | BMP4 | MTTP |
| TCF7L2 | TLR6 | VDR | CPQ | NFU1 |
| GADD45G | FMO4 | BBC3 | AREG | HK1 |
| PIK3R2 | ATG5 | POU5F1 | SIRT6 | MIR214 |
| JAZF1 | FADD | BCL2A1 | IL11 | SELENOT |
| MAP3K11 | CYP4F2 | MIR24-2 | ASPA | ROCK1 |
| NEDD8 | GAA | ATP5PD | PEPD | CAPN2 |
| PTPRC | CYP20A1 | MKI67 | SORL1 | MIR210 |
| SORD | MUC5AC | ALDH3A2 | CHCHD2 | PLD1 |
| C5 | MIR222 | TNFRSF11B | TNFRSF10A | FABP1 |
| PFKM | PYCR2 | IL6R | SUMO1 | TEK |
| SLC7A1 | FIG4 | GLT8D1 | LBR | CXCL16 |
| OSM | GRIN1 | DYRK1A | MIR181A2 | DNM2 |
| TACO1 | GIGYF2 | C5AR1 | ALDH3B1 | CCR7 |
| MUC1 | CANX | ADH1A | FRZB | EEF2 |
| ZFAND1 | UBQLN4 | PKD1 | MIR200C | TNIP1 |
| SCN4B | IL16 | H3C14 | ARNT | CYP19A1 |
| SDC1 | CACNA2D1 | AQP1 | CDC25C | ACTN4 |
| BSG | LYRM4 | KL | ANXA11 | AVP |
| ABCC3 | MRAP | FANCD2 | FIS1 | PGAM5 |
| CAMKK2 | CFAP410 | VIPR1 | ADRB3 | RTN4 |
| MIR19A | FAAH | GLA | PDE4A | TARS2 |
| CX3CR1 | NOD2 | SFTPB | LANCL1 | CD38 |
| FECH | TPPP3 | IL1RAPL2 | TP53INP1 | ENC1 |
| PEX5 | IKBKG | FYN | ABCG2 | MIR199A1 |
| UCN2 | CD46 | KIAA0319L | MIR184 | CXCL2 |
| GPX5 | CD28 | YAP1 | GADD45B | EIF2AK4 |
| NEK1 | HDAC9 | ACAD8 | MIR203A | ADAM10 |
| MT-CO3 | RAG2 | C3 | HRH2 | VEGFC |
| SLC8A1 | MRPS34 | FCGR3A | IL12B | CIITA |
| SIAH1 | MIR23B | SET | MIR92A1 | MMD |
| ITGA2 | TLR5 | PHYH | HSPG2 | PRDM10 |
| RBP4 | DYNC1H1 | HPRT1 | SPARC | SELENOK |
| MAPK8IP1 | TFEB | TAT | HDAC2 | VNN1 |
| MIR142 | GDF15 | SORCS2 | MIR152 | SELENOP |
| ADSL | NEFL | HPX | OTC | LCAT |
| RYR3 | ISG15 | MAP3K1 | BCR | SLC25A1 |
| EPHA4 | MAPKAPK5 | ACTG1 | CLIC1 | SLC7A11 |
| TLR8 | NCF4 | DGKQ | IFNAR1 | MMP8 |
| CDKN2B | TOP1 | ACO2 | MIR25 | CUL3 |
| DUOX1 | CAMK4 | CUL1 | CYB5A | IGF2BP2 |
| UBE2D2 | FAM120A | AQP4 | PDGFB | MIR27A |
| KCNE2 | HAMP | BACH2 | PPP5C | AURKA |
| TLR7 | PTPA | WRN | TTPA | RARA |
| UCN | PKP2 | KCNT1 | ATXN8OS | PARK10 |
| PARK16 | PARK21 | MGMT | LIN28B | SMPD1 |
| GRM1 | DMPK | PYCR1 | UTRN | MIR148B |
| FOXJ1 | MIR144 | CCR5 | SLC40A1 | ITGB3 |
| CCL11 | MECOM | MIR20A | DUSP19 | VASP |
| HAO1 | MIR9-1 | FH | CYP21A2 | CACNA1A |
| BRAF | NOSIP | KLRK1 | ESR2 | PIK3C3 |
| VTN | IL33 | CXCR1 | PRKCG | TALDO1 |
| DECR1 | NR1H2 | HBEGF | AKAP9 | MIR34C |
| MSR1 | RPA1 | RCAN1 | FTH1 | CYP11A1 |
| CHGA | PLCB1 | SENP3 | RRM2B | PDK1 |
| SNCB | MIR107 | MIR181C | CAPN3 | RNASE3 |
| PRPH | CFI | LAMP1 | BLOC1S1 | PML |
| CCK | TIMP2 | TRAF2 | STIP1 | NOSTRIN |
| MYLK | GSTM5 | TPK1 | SLC2A4 | HLA-A |
| UNG | IL3 | RORA | MDH1 | RUNX2 |
| SCARA3 | PDIA3 | DDC | GSTM4 | PIGA |
| BRCA2 | UNC13A | VPS13C | TNFSF11 | CYP2C8 |
| SERPINF1 | DYSF | PSIP1 | TPI1 | PDGFRB |
| CAMK2G | EPHX2 | CR1 | CYP3A5 | AGRN |
| NR2C2 | XRCC6 | PPP3CA | UBQLN1 | FOXO4 |
| MAPK7 | NES | FTL | BACH1 | SCGB1A1 |
| FMO2 | APC | MYD88 | HERPUD1 | CYP17A1 |
| TYRP1 | XRCC5 | FKBP1B | MMP14 | TMEM161A |
| ALOX15 | SOX2 | ASL | CCNF | DIABLO |
| MMP7 | TRPV4 | PGK1 | NDUFA1 | IL23A |
| ATXN1 | JUNB | HSPA14 | CD86 | GAL |
| FGF7 | SCP2 | BCL6 | SULT1A3 | GADD45A |
| GSN | MDH2 | IL2RB | ABCC8 | PPIG |
| MCU | MLYCD | CHMP2B | UBC | MSN |
| PTPN3 | NTF4 | SLC19A3 | CCNB1 | FMR1 |
| HSPA6 | HK2 | SELL | PRKCZ | PTGIS |
| ZC3H12A | AHR | GLS | PPOX | NCAM1 |
| RETN | B2M | CD55 | VKORC1L1 | SLC25A27 |
| EDNRB | PF4 | SMAD2 | SOCS1 | BLK |
| PYGM | CDK6 | MICB | PPARD | UBQLN2 |
| HYOU1 | TACR1 | BDKRB2 | ADRB1 | CTTN |
| DEPDC5 | MIR106B | NQO2 | ABCC2 | SREBF1 |
| SERPINH1 | H2BC21 | LPA | VCL | MIR145 |
| MIR143 | FASN | CASP4 | CD34 | KRT18 |
| CTSG | SLC11A2 | ODC1 | TNFRSF10B | CHEK1 |
| SFTPD | CD69 | PDLIM4 | GH1 | LPL |
| HNF1A | SCARB1 | PTX3 | RAD51 | EZH2 |
| COX15 | LGALS1 | GRIN2A | VAPB | ELAVL1 |
| HRH1 | ATR | PNKP | GFER | CDH5 |
| MIR24-1 | ABCA1 | DAXX | CDH2 | GZMB |
| CALB2 | MUTYH | ERCC8 | DDAH1 | NDUFS7 |
| NDUFS6 | ERO1A | DSP | NDUFA10 | SUMO2 |
| NRAS | IGF2BP1 | CXCL9 | SLC18A2 | PIK3C2A |
| BCL2L11 | STK4 | HNF4A | ALOX12 | GLS2 |
| CFH | F8 | DNAJB1 | TBK1 | ITIH4 |
| LRPPRC | XRCC1 | TP73 | ANGPT2 | AOX1 |
| NEAT1 | NME1 | DES | TRPA1 | TGFA |
| MAP3K7 | EPX | PLG | INSR | RHOD |
| GP1BA | LGALS3 | CYC1 | BAG3 | PARK12 |
| STAT4 | NR1H4 | DNAH8 | MT-ND6 | NDUFA9 |
| FCGR2B | CSK | STK24 | AR | GYG1 |
| PRKD2 | PPIA | CDK4 | TPT1 | COA8 |
| IRF5 | F5 | SCO2 | PKM | GLO1 |
| MYH6 | MME | FOXM1 | ACOX2 | JAK1 |
| H19 | NDRG1 | PEX11B | ERBB2 | FKBP5 |
| MIR133B | TPM1 | MIR221 | VHL | IL2RA |
| MSH2 | C4A | RAB5A | PLAUR | NEIL1 |
| SPR | LCK | MSRB3 | GLE1 | TAF15 |
| GFM2 | SETX | ADAMTS13 | GHRL | KCNMA1 |
| MIR125A | H2AX | FOXP3 | KRT8 | PLA2G2A |
| BRF2 | MIR200B | FKRP | ADPRS | H4-16 |
| ALAD | MIR181A1 | MT-ND4 | NDUFB9 | IGF2 |
| ACTN2 | ISCU | XIAP | CFLAR | GSTA2 |
| NLRP3 | QDPR | CASP2 | SRF | PKLR |
| TYMP | SCO1 | PTS | THBS1 | SYP |
| TYK2 | GRN | OPTN | GRIA1 | PIK3CB |
| IKBKB | CEBPB | CD80 | C4B | PPP1R15A |
| ACSL4 | EIF2B3 | PLA2G4A | TUBA1B | STK11 |
| MMP3 | GRM5 | DRD3 | SMARCA4 | NTRK1 |
| ECE1 | LYN | ELK1 | ALDH3A1 | PDCD1 |
| CCS | SIL1 | IL5 | PSEN2 | ARG2 |
| IDO1 | DYNLL1 | MTR | PDIA2 | SLPI |
| SLC1A1 | EIF2B4 | ADAM17 | CAMP | CYP2A6 |
| HDAC1 | CD79A | CYP27A1 | RXRA | PTPN1 |
| EP300 | CXCR3 | NAT2 | EDNRA | PFN1 |
| MT-ND3 | NTHL1 | KCNJ2 | NRG1 | CHCHD10 |
| BECN1 | CLEC4A | COQ2 | EIF4EBP1 | SGK1 |
| ANGPT1 | SOCS3 | UCP1 | MIR93 | AIF1 |
| IL15 | TRIM21 | LTA | NAMPT | MIR29A |
| MFN2 | CALM2 | TIA1 | RELA | NGFR |
| TRAP1 | HGF | GAP43 | MIR433 | SLC6A2 |
| CD4 | MYO9A | PENK | EPRS1 | CDH1 |
| CFTR | RPS6KA5 | PVALB | MRPL44 | CTSB |
| NTS | ERBB4 | EIF4G1 | KLF4 | CYP2B6 |
| ACE2 | SESN1 | NRF1 | PECAM1 | KLF2 |
| GSS | EIF2AK1 | FLT1 | RPS6KB1 | OXA1L |
| ATXN3 | HLA-B | ATF3 | RAF1 | MALAT1 |
| ADA | MAPK11 | GRIN2B | ANXA2 | COX6B1 |
| HMGCL | CXCR4 | CXCL10 | MMP13 | MIR122 |
| STK39 | GCLM | TLR3 | BAD | TBP |
| CCN2 | TJP1 | ALDH9A1 | BIRC5 | FCGR3B |
| PDGFRL | NLRP1 | KCNE1 | GAD1 | ENO2 |
| PEX12 | CDKN1B | GJA1 | HSPA1B | LOC111365141 |
| FGF1 | DRD4 | IRF1 | ATF2 | CDK1 |
| RNF112 | HMGCR | DHCR24 | NTRK2 | CXCL1 |
| AHSP | NDUFA6 | PLCG1 | IRAK1 | FMO1 |
| HSP90AB1 | ITGAL | AKT2 | FDXR | MIR126 |
| CCR6 | KIF1B | DSPP | CCNA2 | CPOX |
| ALDH1A1 | HSPB2 | CNTF | MT3 | SDHAF2 |
| EPAS1 | LONP1 | ABCC1 | SETD2 | E2F1 |
| MTFMT | CALB1 | MBP | S100A9 | NDUFS1 |
| IGF1R | CHUK | DNASE1 | IFNB1 | VIM |
| ANK2 | MAP2K7 | CR2 | DLG4 | DRD1 |
| PCNA | ADCY10 | PLAT | TSC1 | ELN |
| MBL2 | BMP2 | LCN2 | GLUD1 | TNFSF10 |
| DMD | MCL1 | NOL3 | CST3 | CBS |
| NDUFS8 | CHKA | DDAH2 | NTF3 | ACTB |
| PRKAB1 | STUB1 | FCGR2A | ITGB2 | MATR3 |
| APOB | STK25 | TLR9 | CSF2 | APAF1 |
| PDYN | OPRD1 | TNFAIP3 | ABCB1 | TGM2 |
| ACTA1 | MIR23A | GRB2 | HSD17B10 | MRPS14 |
| MIR132 | LTF | CCL4 | NDUFS2 | BTD |
| IFNA1 | RB1 | SMAD4 | MAP2K6 | SRXN1 |
| LPO | HBA1 | IRS1 | SLC17A5 | KCNQ1 |
| HTR2C | ENO1 | LAMP2 | ITGB1 | SERPINA1 |
| S100B | LOC110973015 | REST | PIK3R1 | CDC42 |
| CXCL12 | DAO | BAK1 | HCRT | ADORA2A |
| MTHFR | CSF3 | BRCA1 | GNAS | SIGMAR1 |
| IREB2 | TGFBR1 | CARS2 | CALM3 | MAP2 |
| MIF | CTSD | MAPKAPK3 | PAH | OXR1 |
| CACNB4 | PXN | HTR3A | IGF2R | CASP7 |
| GSTO2 | ANG | EPHA3 | PTPN22 | MRPS16 |
| GLUD2 | CREBBP | SLC25A13 | SIRT3 | GSTA4 |
| IDH2 | TOR1A | DCTN1 | MIR17 | C12orf65 |
| EPHX1 | TGFBR2 | BCHE | ERCC6 | NFE2L1 |
| EIF2AK2 | ADM | H2AC18 | COL2A1 | TRPV1 |
| VIP | SYK | A2M | ALOX5 | CD44 |
| IAPP | PRKCA | SNCAIP | S100A8 | NAGS |
| HNRNPA1 | ATP13A2 | SLC25A3 | DAPK1 | TTR |
| EIF4E | DLST | CRYAA | UBE2L3 | NEFH |
| G3BP1 | ADH1C | DNMT1 | MRPS22 | TFAM |
| OSGIN2 | CCL3 | SCN4A | UCHL1 | MIR195 |
| FGFR1 | OXT | MIR34A | AKR1A1 | OPA1 |
| VARS2 | SLC1A2 | CRHR1 | POR | PRKG1 |
| TGFB3 | KCNJ5 | BACE1 | SNTA1 | DRD5 |
| SERPINA3 | HSPA9 | SCN2A | AMPD1 | TNFRSF1B |
| ITPR1 | TSFM | MT-TK | MIR223 | TGFB2 |
| UCP3 | IDH1 | CSF1 | DNM1L | MET |
| TSC2 | PLA2G6 | PGD | KCNH2 | HMOX2 |
| PPIF | EHHADH | TXNRD2 | AKR1B1 | GSTM2 |
| MAP2K3 | NGB | MMP1 | NCF1 | MECP2 |
| NPPB | SIRT2 | STAT1 | ECHS1 | AOC3 |
| H6PD | FGF2 | GPX2 | GLRX2 | OSER1 |
| GSTM3 | KRIT1 | SDHAF1 | OPRM1 | SESN2 |
| APOH | VDAC1 | REN | SST | ADRB2 |
| NR3C2 | GSK3B | KRAS | MYC | ADH5 |
| HTR1A | FXN | NPPA | PTK2 | CPT1B |
| HLA-DRA | UGT1A1 | PRKD1 | CYB5R3 | GPX4 |
| GPT | FMO3 | F3 | TFRC | JAK2 |
| MT-TL1 | IL1R1 | MAP2K1 | MT-CO2 | ATP2A2 |
| PLA2G7 | CD40 | MAP2K4 | GSTO1 | TPH1 |
| BCL2L1 | ACHE | CRAT | GCLC | NFKBIA |
| SGCB | IL4 | UCP2 | NR4A2 | CD40LG |
| IL17A | EGR1 | LOC110806262 | TRMT10C | PNPT1 |
| CDKN2A | PTK2B | CCL5 | NPM1 | ETS1 |
| PRODH | ACO1 | EEF1A1 | OGDH | TAC1 |
| CASP1 | CYP11B2 | NDUFAF2 | PRKCD | SLC5A7 |
| SMAD3 | ENG | GLUL | HSD17B4 | RAC2 |
| MT-ND2 | PON3 | ATXN2 | PTEN | TSPO |
| ELANE | SDHC | TUFM | ERN1 | POMC |
| DBH | CYP2C9 | GPX7 | P4HB | FUS |
| MT-ND5 | FASLG | HLA-DRB1 | MIR155 | TF |
| CHKB | CDK2 | CD36 | HFE | ACP1 |
| GSTA1 | NOTCH1 | DUSP1 | PRDX4 | PTPN11 |
| AGTR1 | HSP90B1 | SLC18A3 | FN1 | PRKCB |
| CNR1 | TREM2 | NDUFV2 | GLRX | ITGAM |
| TECRL | IL1RN | CACNA1S | HTT | PLAU |
| TIMP1 | CDK5 | CALR | MGST1 | SUOX |
| PTGS1 | CYP2C19 | TREX1 | TTN | DLD |
| ADCYAP1 | CS | TXNIP | IL18 | GCDH |
| APOA1 | CYGB | CCND1 | PDHA1 | ASS1 |
| LRRK2 | HTR2A | DRD2 | SLC6A3 | MAPKAPK2 |
| ARG1 | GGT1 | SLC25A4 | NPY | GBA |
| BMP6 | HSPD1 | CYP1B1 | NOX1 | HP |
| LEP | BLVRB | NOS1AP | FARS2 | HRAS |
| CALCA | RAC1 | SNAP25 | PRKAA2 | PRKAA1 |
| EPO | MSRB2 | LOX | NDUFB8 | PDE5A |
| CRYAB | EGFR | RPS27A | MDM2 | THBD |
| NDUFV1 | CTLA4 | MIR22 | MAPK9 | NGF |
| IL2 | SRC | CREB1 | ATF6 | MIR146A |
| TLR2 | ATM | SDHD | IL1A | NDUFS3 |
| MT-ATP6 | MT-CYB | CDKN1A | OSGIN1 | SLC1A3 |
| ATF4 | GTPBP3 | RHOA | PPARA | ABL1 |
| KDR | SPP1 | ACAD9 | AGT | TRPM2 |
| GSTT1 | PIK3CA | PRDX1 | HMGB1 | CLU |
| PIK3CG | CDKN3 | HBB | NDUFA12 | SELP |
| ATP5F1A | SERPINE1 | C1QBP | PRDX3 | TXNRD1 |
| SLC22A5 | MSRB1 | AGER | PRL | LDLR |
| CTNNB1 | TRDN | CASQ2 | PC | CALM1 |
| ETFB | SHC1 | COMT | ANXA5 | MMP2 |
| TH | SELE | STAT3 | NUDT1 | MT-CO1 |
| MIR21 | EIF2S1 | EIF2AK3 | TNFRSF1A | TERT |
| IL13 | GDNF | SLC2A1 | FAS | SOD3 |
| MB | MT-ND1 | SDHA | CYP1A2 | SDHB |
| NR3C1 | MMP9 | TXN2 | OXSR1 | HSPA8 |
| C9orf72 | GFAP | IGF1 | TPO | EGF |
| MYH7 | MSRA | CYP2E1 | GCH1 | ELAC2 |
| MAOB | PRDX6 | CYP1A1 | PINK1 | GPX3 |
| ACADL | SP1 | NOX4 | CASP9 | F2 |
| ETFA | PPARG | HTRA2 | ADIPOQ | CYP3A4 |
| ALDH2 | FOXO3 | COX5A | SELENON | OGG1 |
| KNG1 | MTOR | CHAT | ABCD1 | MTO1 |
| TLR4 | BAX | PRDX2 | POLG | MAPK3 |
| VCAM1 | HSF1 | NCF2 | PRNP | FOS |
| MAOA | APOE | HIF1A | CACNA1C | CASP8 |
| LMNA | XBP1 | CRH | PPARGC1A | CAV1 |
| PON2 | BCL2 | TARDBP | MAPK10 | HSPA1A |
| ACE | APEX1 | OLR1 | ESR1 | MAP3K5 |
| VCP | AIFM1 | ICAM1 | CP | SCN5A |
| HSPB1 | BDNF | NFKB1 | PRDX5 | HBG2 |
| CAV3 | TYR | KEAP1 | GSTM1 | AARS2 |
| FOXO1 | PSEN1 | DDIT3 | GSTP1 | CPT1A |
| SLC25A20 | GPX1 | SQSTM1 | ETFDH | HSPA5 |
| ACOX1 | CYP2D6 | VWF | NDUFS4 | GAPDH |
| MAPT | PARP1 | VEGFA | RYR2 | CCL2 |
| HADHB | RYR1 | CYBB | GFM1 | HSPA4 |
| PTGS2 | SNCA | IFNG | NQO1 | HSP90AA1 |
| EDN1 | TGFB1 | JUN | CYBA | CXCL8 |
| CRP | ALB | SIRT1 | ACADS | HADH |
| AKT1 | INS | IL10 | ACADVL | TXN |
| CASP3 | G6PD | MAPK1 | SLC6A4 | ACADM |
| IL1B | CYCS | MAPK8 | HADHA | PRKN |
| IL6 | PON1 | PARK7 | GSR | XDH |
| APP | MAPK14 | MPO | SOD2 | CPT2 |
| NFE2L2 | TP53 | NOS1 | HMOX1 | TNF |
| CAT | NOS2 | SOD1 | NOS3 |  |
