# Supplementary figures and images for: Comprehensive analysis of prognosis of cuproptosis-related oxidative stress genes in multiple myeloma
Source: Front Genet. 2023 Mar 31;14:1100170. doi: 10.3389/fgene.2023.1100170 (PMC10102368; doi:10.3389/fgene.2023.1100170)

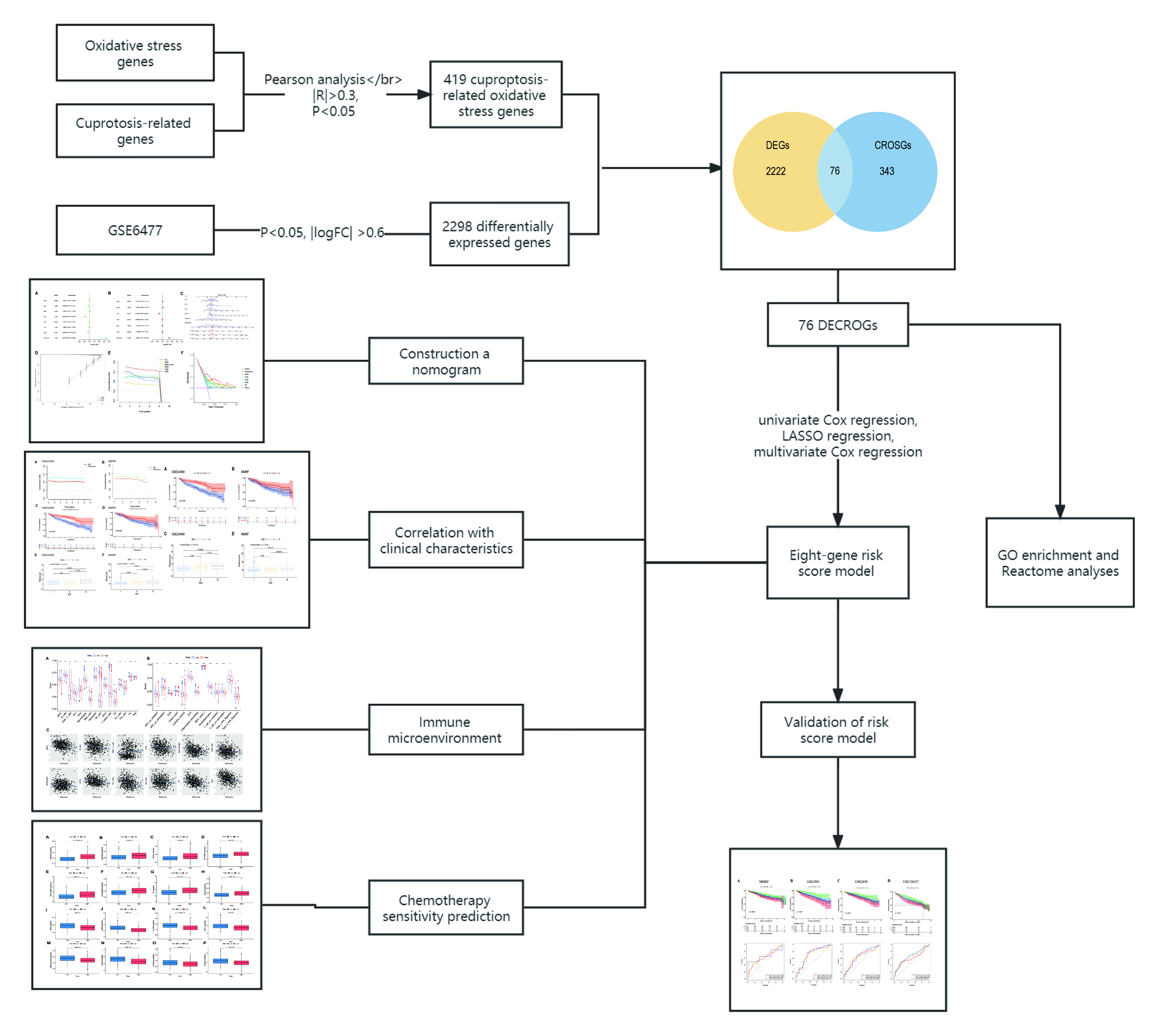

Supplement: Supplementary file 2 [file DataSheet2.ZIP › Figure 1.tif]

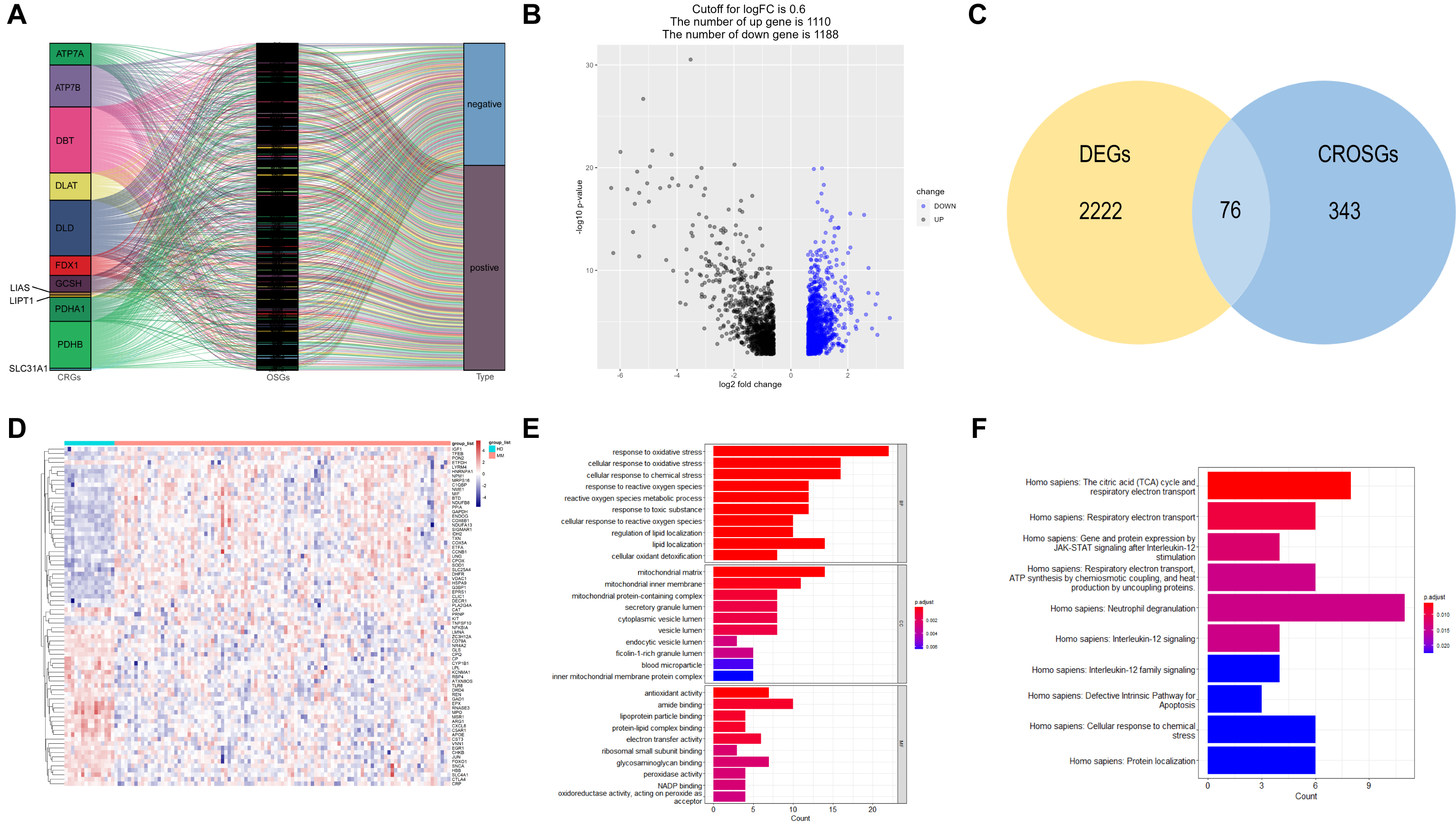

Supplement: Supplementary file 2 [file DataSheet2.ZIP › Figure 2.tif]

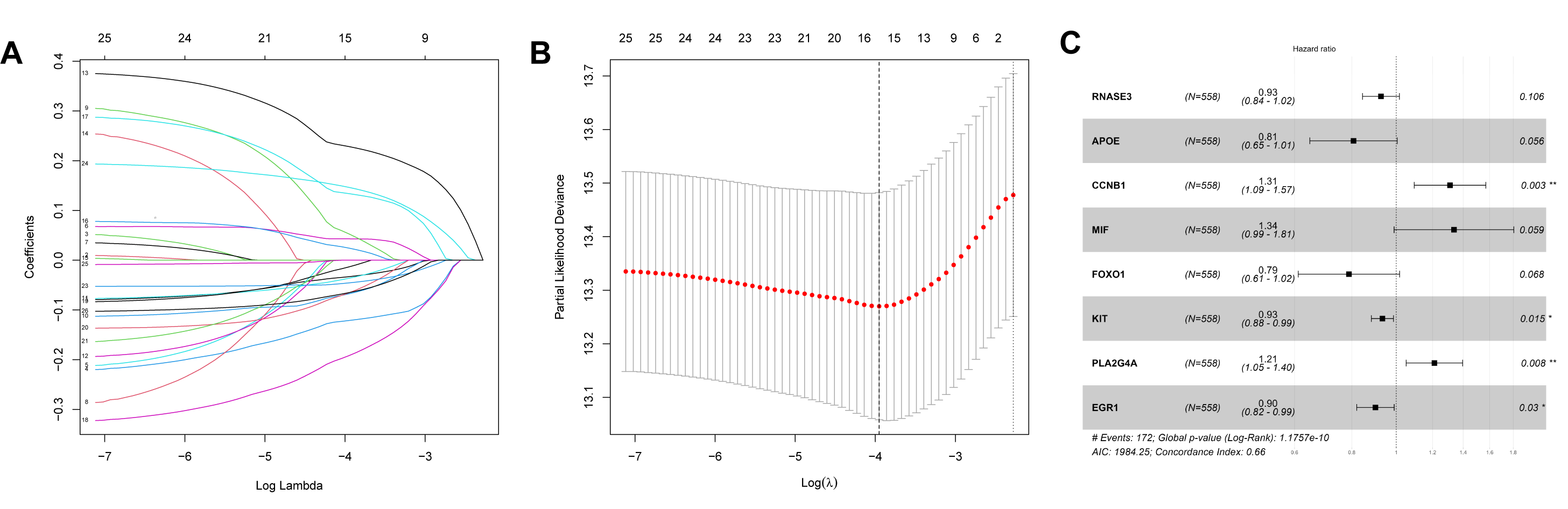

Supplement: Supplementary file 2 [file DataSheet2.ZIP › Figure 3.tif]

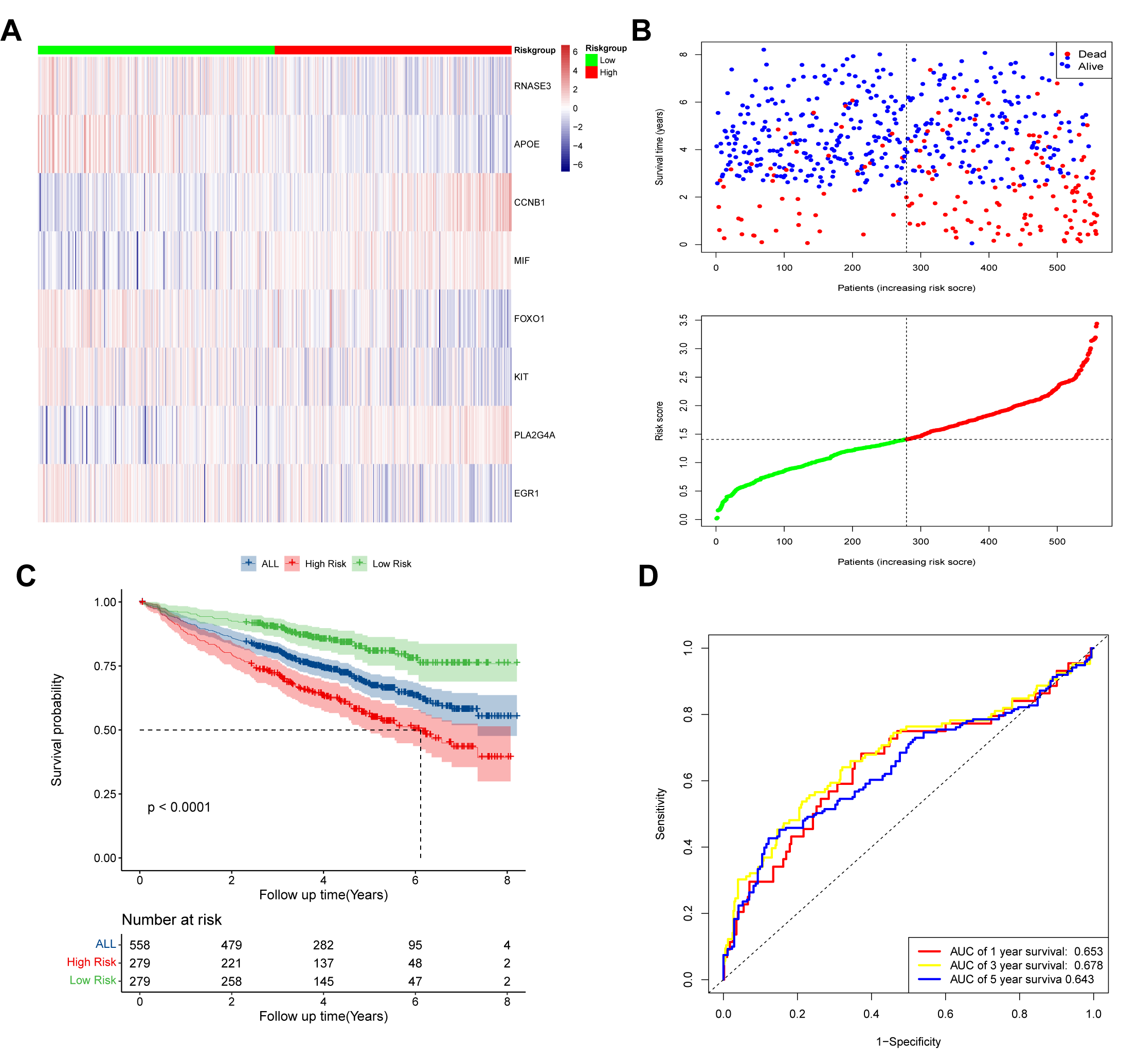

Supplement: Supplementary file 2 [file DataSheet2.ZIP › Figure 4.tif]

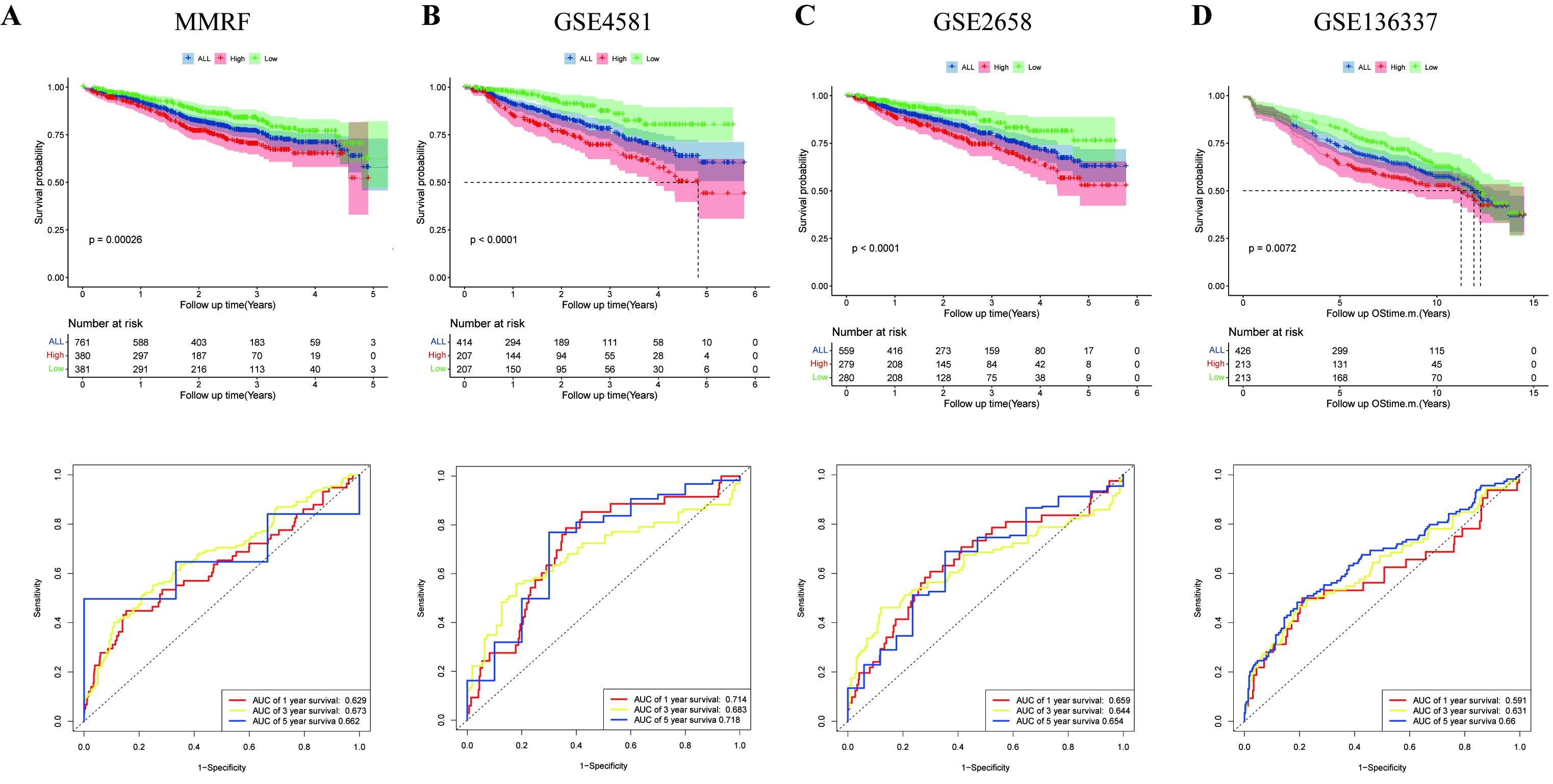

Supplement: Supplementary file 2 [file DataSheet2.ZIP › Figure 5.tif]

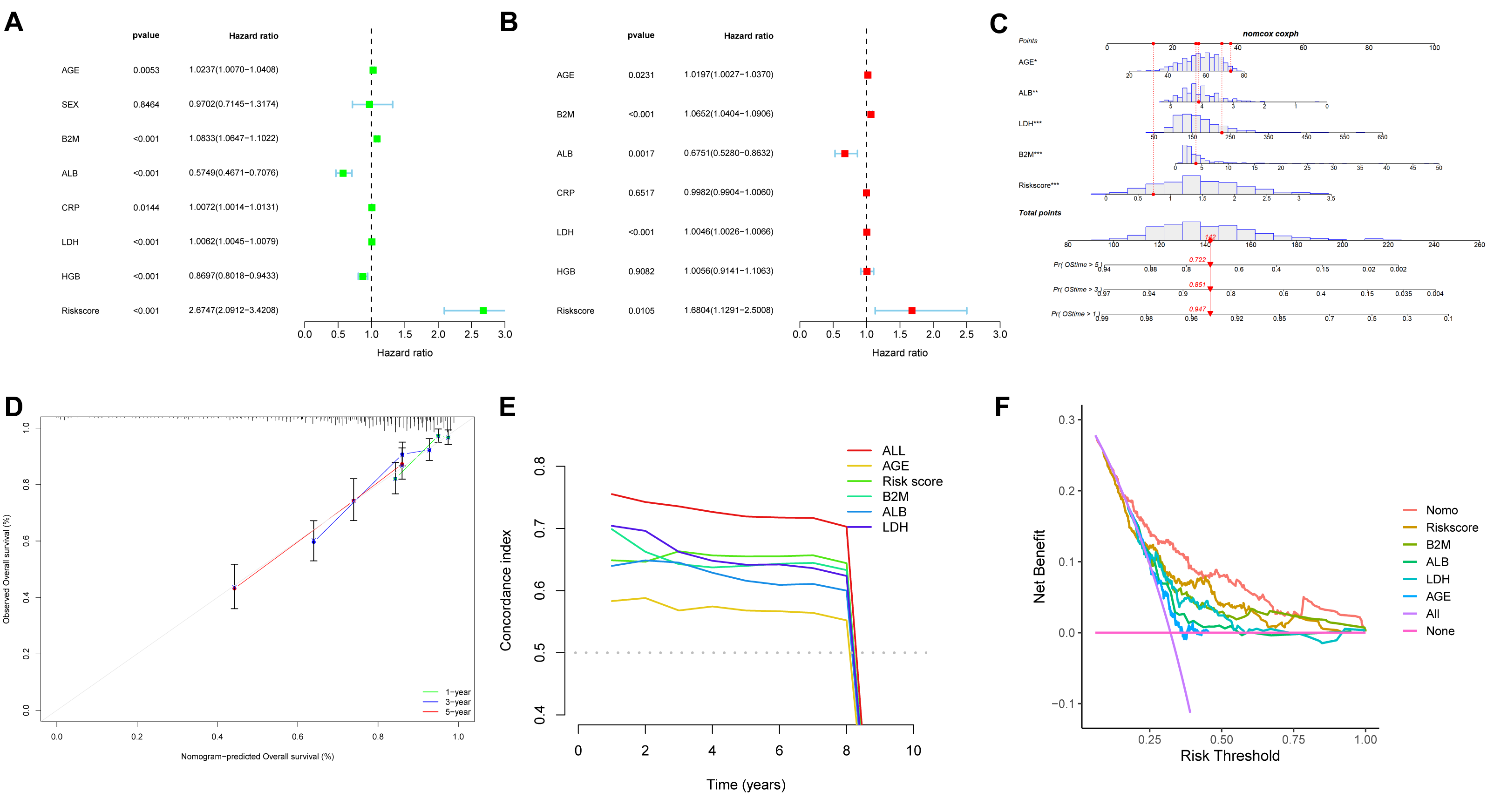

Supplement: Supplementary file 2 [file DataSheet2.ZIP › Figure 6.tif]

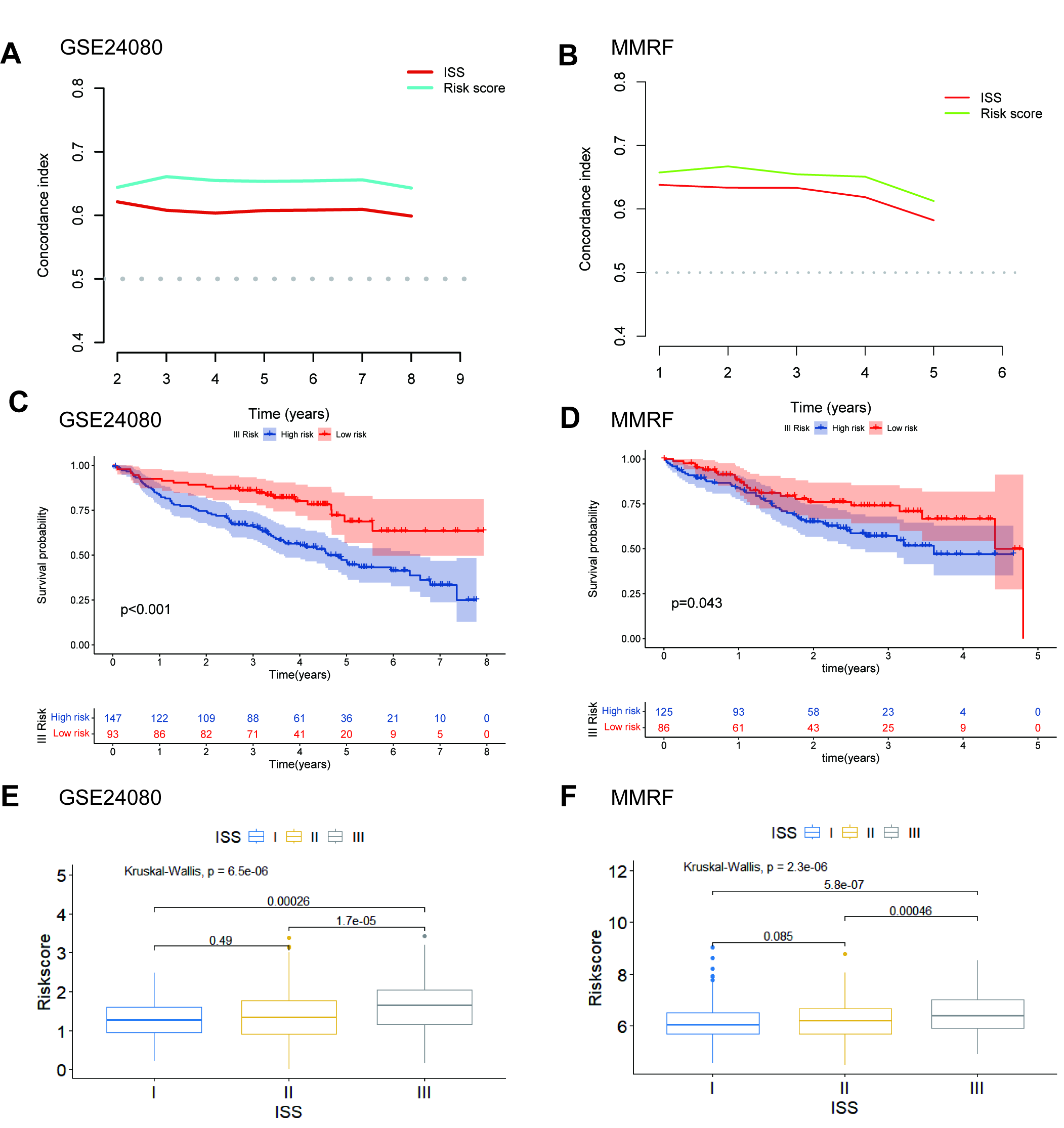

Supplement: Supplementary file 2 [file DataSheet2.ZIP › Figure 7.tif]

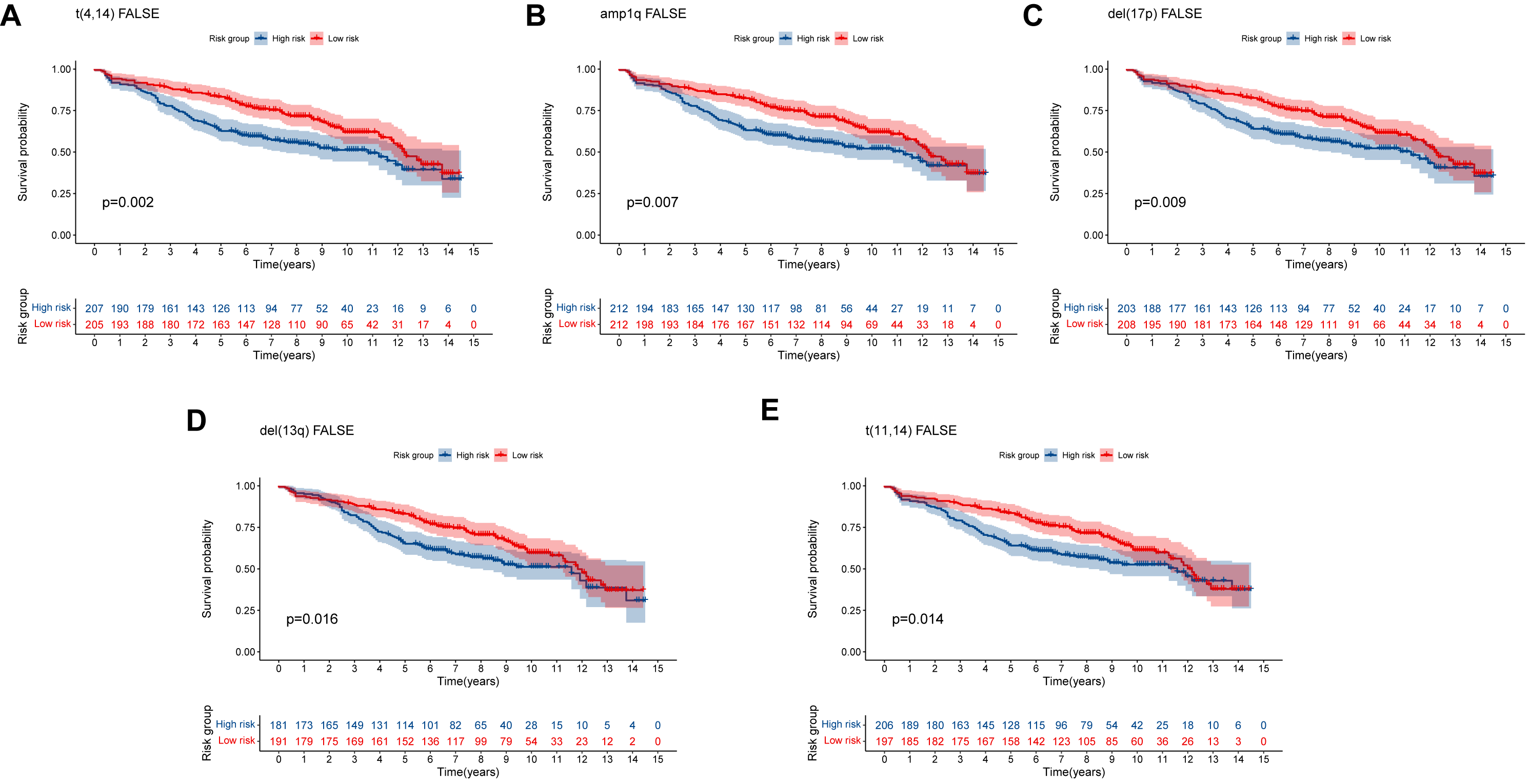

Supplement: Supplementary file 2 [file DataSheet2.ZIP › Figure 8.tif]

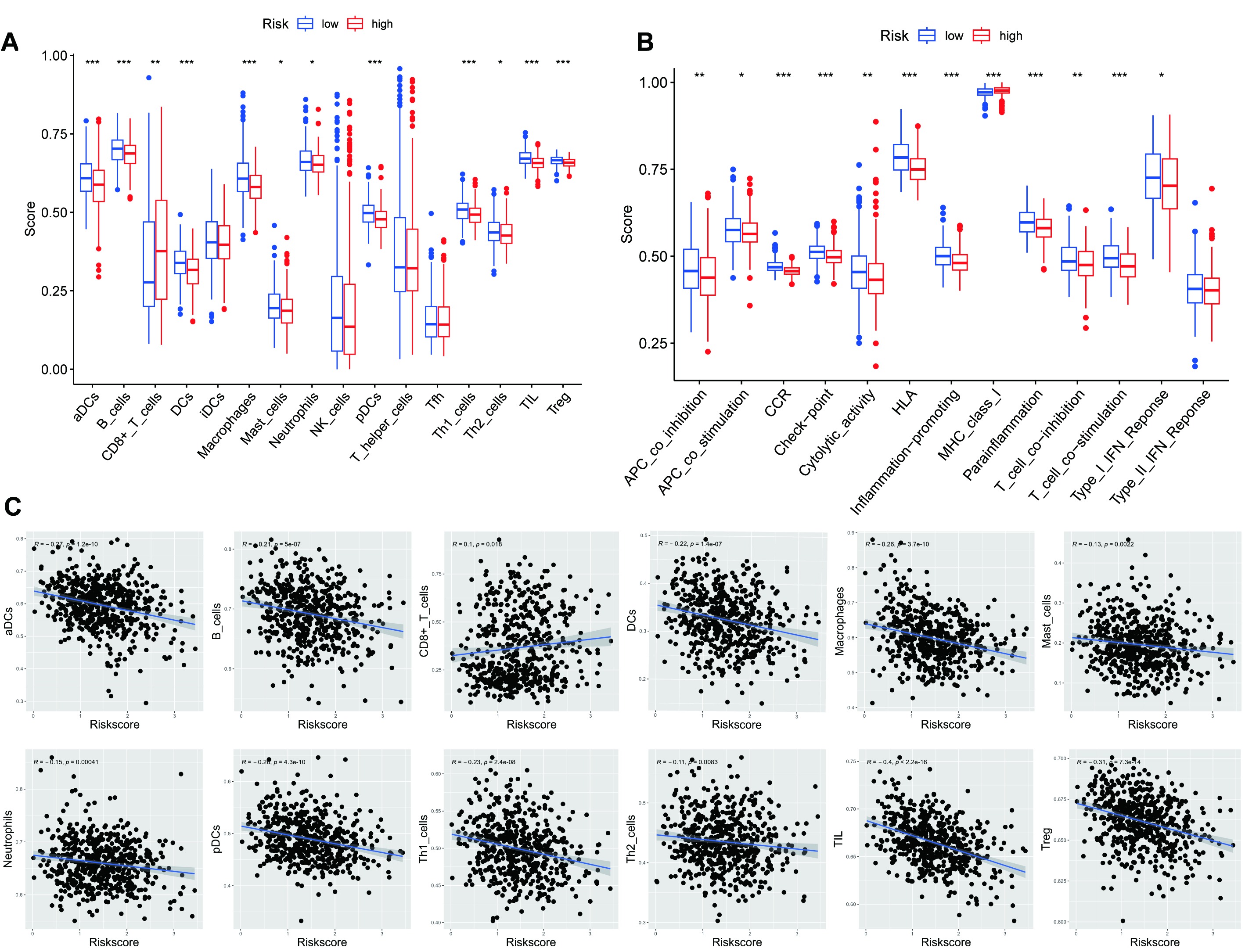

Supplement: Supplementary file 2 [file DataSheet2.ZIP › Figure 9.tif]

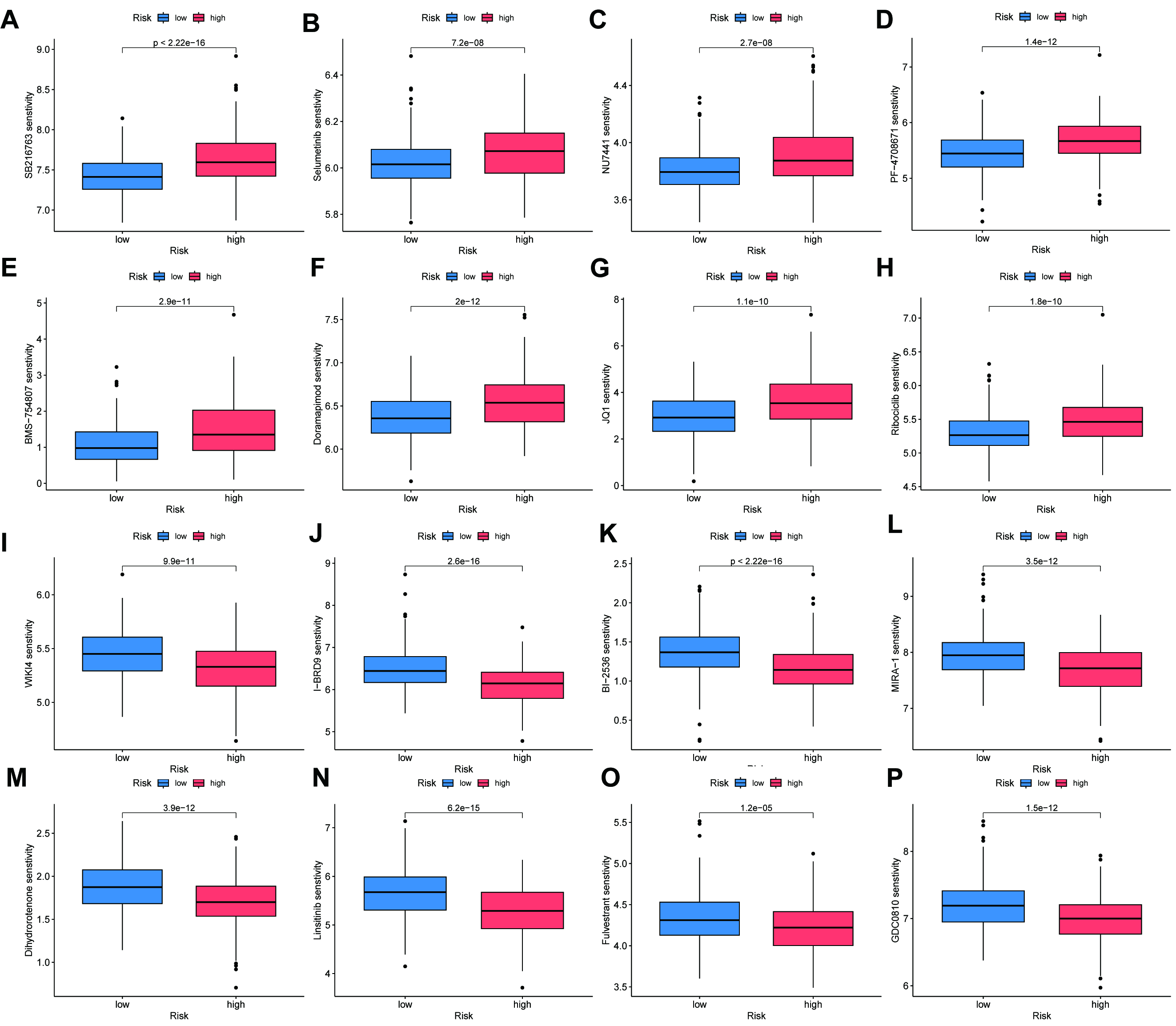

Supplement: Supplementary file 2 [file DataSheet2.ZIP › Figure 10.tif]

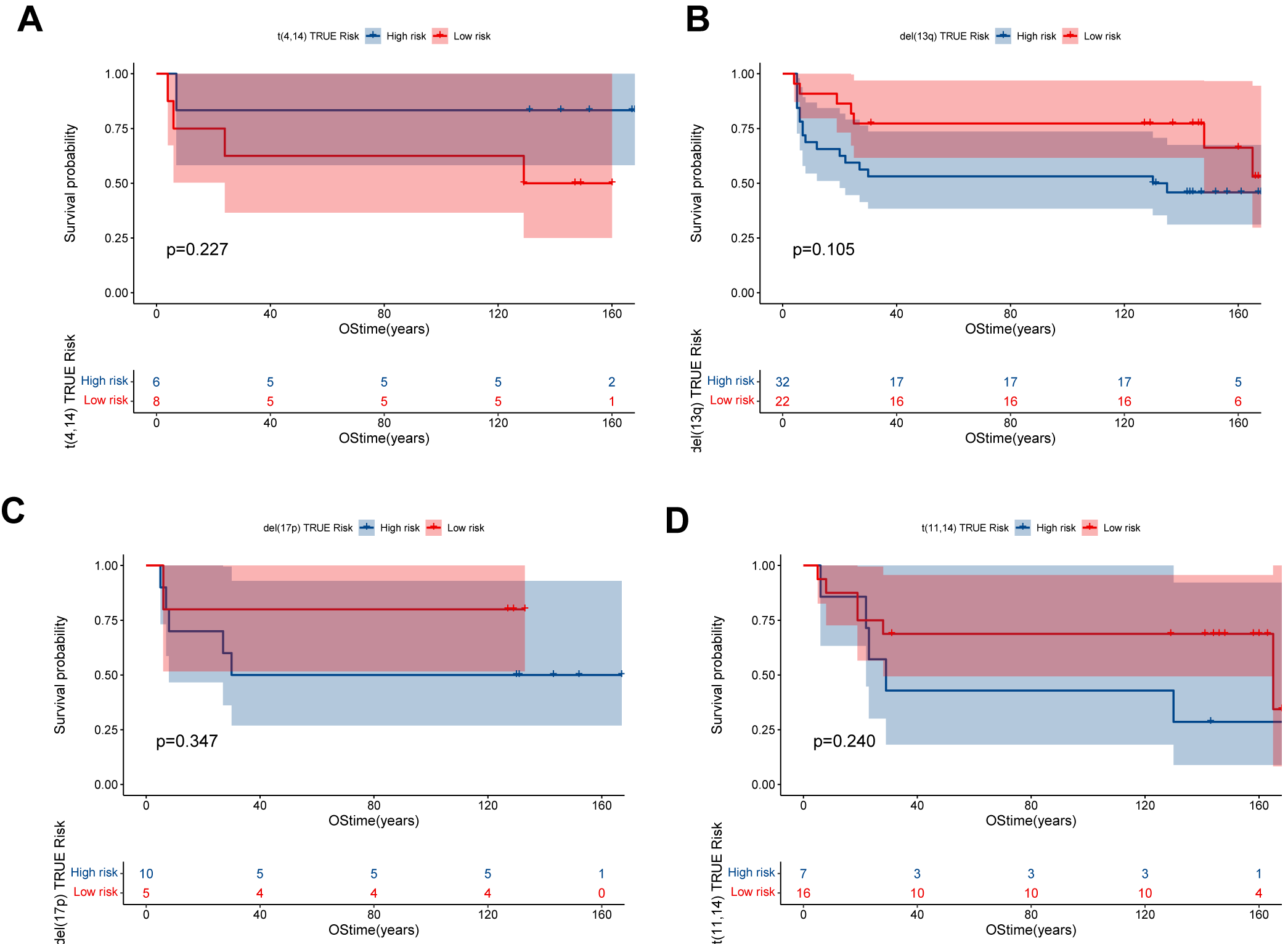

Supplement: Supplementary file 2 [file DataSheet2.ZIP › Supplement Figure 2.tif]

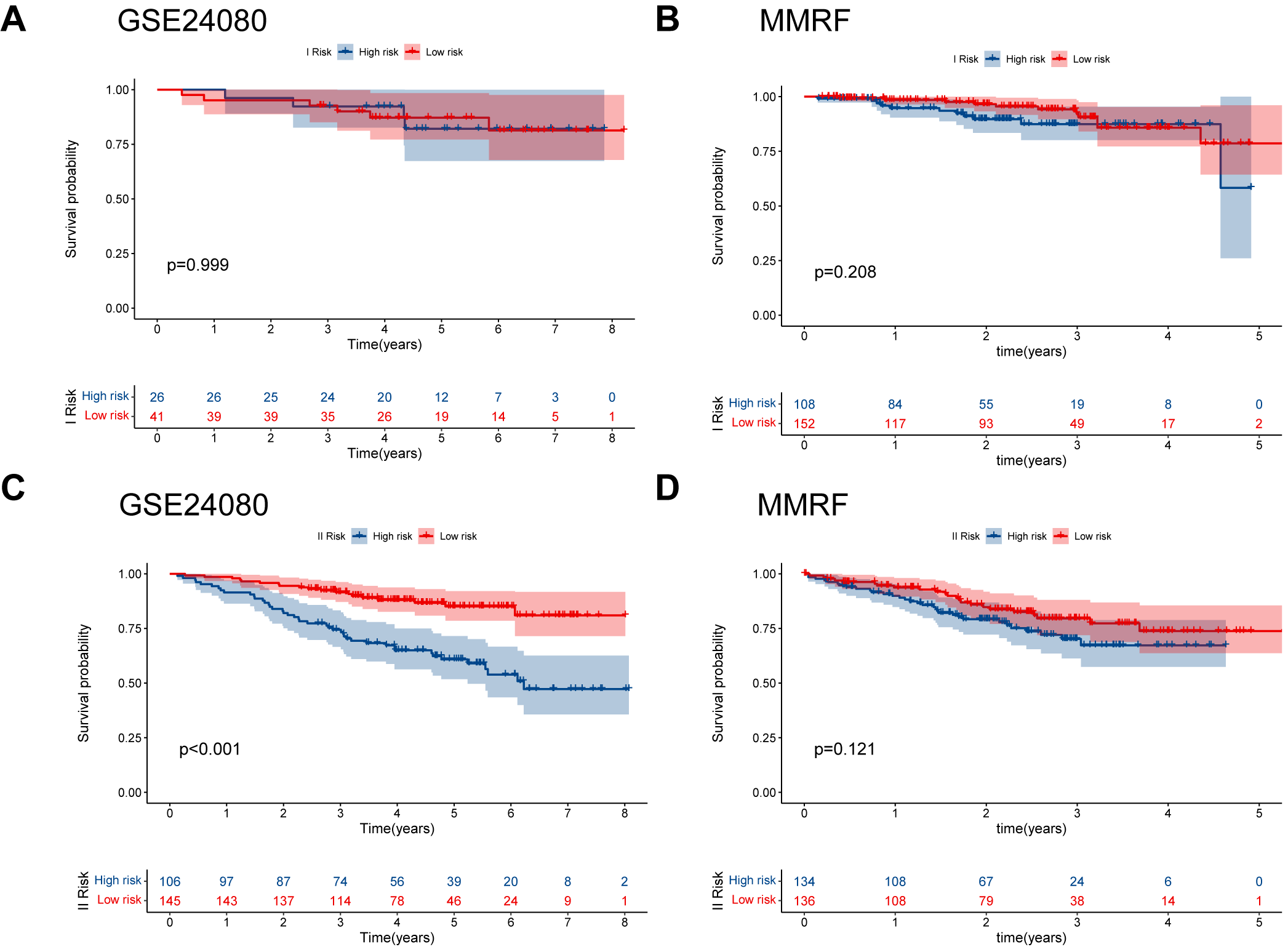

Supplement: Supplementary file 2 [file DataSheet2.ZIP › Supplement Figure1.tif]
